# Ecological theory sheds light on plasmid diversity and dynamics

**DOI:** 10.1371/journal.pbio.3003918

**Published:** 2026-08-28

**Authors:** Rémi Tuffet, Emma Acacia, Charles Coluzzi, Xavier Charpentier, Thomas Koffel, Samuel Venner

**Affiliations:** 1 Laboratoire de Biométrie et de Biologie Évolutive, Evolutionary Ecology Department, Université Claude Bernard Lyon 1, Villeurbanne, France; 2 Centre International de Recherche en Infectiologie, Bacteriology Department, Université Claude Bernard Lyon 1, Lyon, France; University of Lausanne, SWITZERLAND

## Abstract

Bacterial genomes are remarkably dynamic, shaped by horizontal gene transfer. Plasmids are key actors in this process, fueling rapid bacterial adaptation to stresses such as antibiotics. Yet, plasmids follow evolutionary trajectories of their own, defying traditional genetic frameworks. Beyond the co-evolution of traits directly involved in plasmid-host relationships, it is now essential to draw from ecological theory to understand plasmid assemblages. By viewing plasmids as ecological entities competing for a shared resource, the bacterial host, we show that their distribution within bacterial genomes mirrors the structure of ecological communities. Our minimal stochastic model, inspired by community ecology, reveals that plasmid diversity arises from the combined action of niche differentiation and neutral processes. These results challenge deterministic views of genome organization, highlighting the central role of stochasticity and drift. This work establishes a theoretical bridge between microbial genomics and ecology, offering a new framework to understand—and potentially control—the evolution of bacterial genomes.

## Abstract 2

Bacterial genomes are remarkably dynamic, shaped by horizontal transfer of plasmids that follow evolutionary trajectories of their own. This study uses eco-evolutionary perspectives to reveal how the diversity of plasmid communities is shaped by competition, drift, innovation and dispersal, contributing to bacterial adaptation and evolution.

## Introduction

The genetic repertoire of bacteria, even within a single species, can be remarkably diverse [1,2]. This observation gave rise to the concept of the pangenome, the full set of genes found across all strains of a species [3,4]. The pangenome captures the genomic flexibility that underpins bacterial adaptation to fluctuating environments [5–7]. Such flexibility has major consequences, including for human health, facilitating the rapid spread of antimicrobial resistance (AMR) and virulence genes in pathogenic bacteria [8–12]. A major driver of this genomic flexibility is horizontal gene transfer (HGT) mediated by mobile genetic elements (MGEs), particularly plasmids [7,13–15].

Plasmids are self-replicating DNA molecules that move between bacterial cells, both within and across species [10,11,13,16]. This capacity decouples plasmids’ evolutionary trajectory from that of their host, while maintaining intimate interdependence [17–20]. By focusing on this essential plasmid-host relationship, a significant research effort has aimed to resolve the “plasmid paradox” [21–24]: the apparent contradiction between the metabolic burden these genetic elements impose on their host and their ubiquitous persistence, even when natural selection at the bacterial level should favor their elimination [25,26].

To explain this maintenance, early models, such as those by Stewart and Levin [27], proposed first that plasmids act as “molecular parasites” whose horizontal transfer rates (HTR) via conjugation surpass both their metabolic costs and their rate of segregational loss. Building on this, recent studies suggest that transfer opportunities between species within complex bacterial communities further promote their persistence [28,29]. Second, the emergence of compensatory mutations—either on the chromosome or the plasmid—can mitigate the fitness cost to the host, facilitating long-term stability [22]. Third, plasmids have evolved strategies to maximize vertical transmission, either through high copy numbers [30] or “addiction” systems (toxin–antitoxin modules) that eliminate any daughter cell losing the plasmid via post-segregational killing [31]. Finally, environmental heterogeneity may allow some mutualistic plasmids to persist as reservoirs of adaptive genes, such as those for antibiotic resistance [32].

Consequently, plasmid traits and their relationships with hosts are remarkably diverse, spanning the continuum from mutualism to parasitism [18,33,34]. Plasmids differ dramatically in size [35–37], copy number, transmission modes [35,36], and accessory gene content [33]. Conjugative plasmids, for instance, display highly variable HTRs shaped by both plasmid traits and host genetic background [38–41]. Their vertical transmission capacity also varies [25,42,43], enhanced by segregation systems or the carriage of adaptive genes [31,33,44–47]. While classical genomics excels at describing this diversity [48,49], it often falls short of explaining how the interactions between plasmids govern the maintenance of such vast plasmid assemblages. Understanding the ecological and evolutionary drivers of this diversity remains a major challenge, shifting the focus from single plasmid-host interactions to the broader dynamics of plasmid communities [19].

Within these communities, plasmids do not interact solely with their bacterial hosts; they also interact with each other [50,51]. They can be viewed as ecological entities competing for a shared resource: the bacterial host, which can only support a limited number of distinct plasmids [52–54]. Some competitive mechanisms, such as surface exclusion or CRISPR-based immunity, are evident and well-documented [54,55]. Other “cryptic” mechanisms, such as indirect competition, likely exert a strong structuring effect on plasmid diversity, much like interspecific competition shapes ecological communities. Unlike traditional genomics, community and evolutionary ecology focus precisely on these questions: how do species interact, compete, and coexist, and how do their traits evolve within these assemblages?

Applying this perspective to plasmids opens a new conceptual space where plasmids are viewed as a community of competing entities. While ecological frameworks have been proposed to understand microbial diversity [56], the dynamics of transposable elements in eukaryotes [57] or MGEs in bacteria [19,58], we argue that a robust, integrative framework is now essential to weigh the relative importance of the various processes driving plasmid eco-evolutionary dynamics.

To address this, we first draw on published data to highlight parallels between the patterns of plasmid diversity within pangenomes and species within ecological communities. We then develop a minimal dynamical model to show how core ecological theories—niche theory, neutral theory, and their unification—can reproduce observed patterns of plasmid assemblages. These frameworks help explain not only why some plasmids dominate while others remain rare, but also how diversity persists despite strong competitive forces. By bridging microbial genomics and ecological theory, we aim to participate in laying the foundations for an ecology of MGEs, revealing the processes that structure their diversity and, ultimately, bacterial genome evolution.

## Results

### Similarities between plasmid and species diversity

Trait variation and similarities among plasmids likely shapes their assemblages, just as trait differences and similarities structure communities of competing species. Although the concept of bacterial species is debated [59], genetic proximity now defines robust taxonomic units [60], providing a powerful framework for the study of bacterial ecology and evolution. Similarly, sequence similarity methods group plasmids into coherent taxonomic units (PTUs [61,62]), allowing PTUs to be treated as ecological species competing for the same resource, the bacterial host.

**Fig 1:**
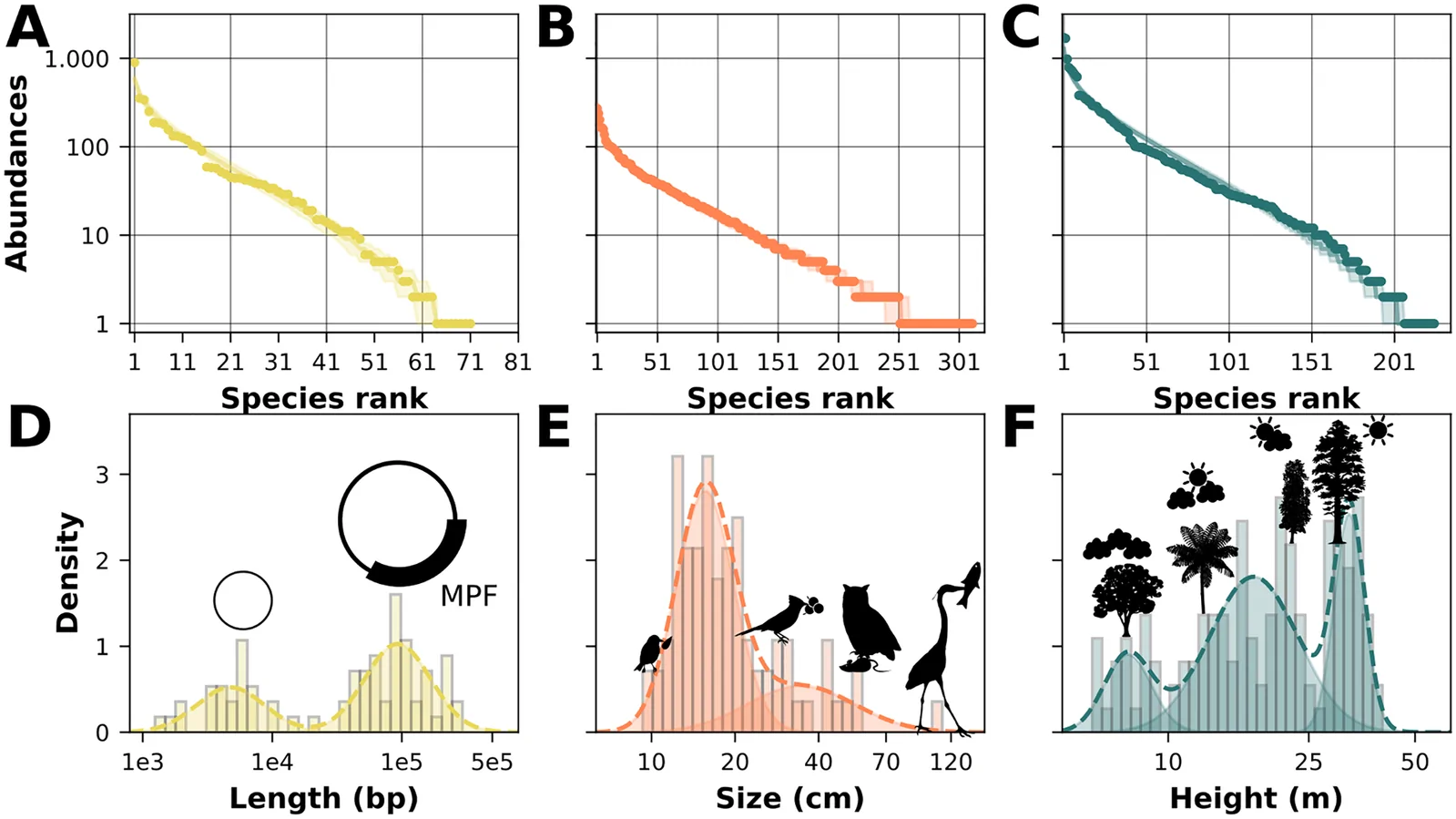
Similar Diversity Structures in Plasmid and Ecological Communities. The first row of panels illustrates rank-abundance curve of **(A)** plasmid species (Plasmid Taxonomic Unit) in *Escherichia coli* genomes (complete genomes from NCBI), **(B)** temperate birds (Wyoming, USA in 2024, GBIF iNaturalist Research-grade Observations), and **(C)** tropical trees (Barro Colorado Island (BCI), Panama; data from [70]). Bird, tree, and plasmid communities all exhibit similar abundance patterns: a few species are highly abundant, many are moderately represented, and a significant number are extremely rare. These distributions were fitted using the Power-bend model (predicted abundances and 95% IC, see Materials and Methods) which consistently outperformed six other classical species-abundance models (S1 Table). Interestingly, similar patterns also emerge from neutral models (see section on Neutral theory for plasmids). The second row of panels illustrates the distribution of **(D)** plasmid length (complete genomes from NCBI), **(E)** body size of temperate birds (North America, data from [71]), and **(F)** maximal tree height in tropical forests (BCI, Panama, data from [72]). Body size is a key trait often used to reflect differences in ecological niches. Just like birds or trees coexisting in their communities, plasmids found in *E. coli* show a great degree of variation in size, ranging from ~1,000 to 300,000 base pairs (Fig 1D). These size differences are associated with functional differences: larger plasmids are typically conjugative, while smaller ones are not [35]. Similarly, variation in bird body size reflects dietary differences [73,74]—from small species feeding on seeds and small insects, to medium-sized frugivorous and insectivorous species, and larger species feeding on larger invertebrates and small to medium-sized vertebrates. In trees, height variation is linked to differences in light sensitivity and shade tolerance [65]. Such trait variation contributes to niche partitioning and is thought to stabilize species coexistence (see section Ecological niche theory for plasmids). Interestingly, the size distributions of plasmids, birds, and trees are often multimodal, which may support recent efforts to unify biodiversity theories (see Unification of biodiversity theories applied to plasmids). We tested the multimodality with a Gaussian Mixture Model (with 1–7 Gaussian) resulting in 2, 2, and 3 mixed Gaussian for plasmids, birds, and trees, respectively (see Materials and methods). The data and code required to generate this Figure can be found in https://doi.org/10.5281/zenodo.21220021.

In this context, plasmid assembly patterns mirror those of competing species within a trophic level. Among the 2,012 nonredundant complete genomes of *Escherichia coli* from NCBI, we identified 4,411 plasmids clustering into 71 PTUs. Their abundances follow a characteristic rank-abundance curve (RAC [63]) where a few abundant plasmid species coexist with many species of intermediate or low frequency (Fig 1A). This distribution closely resembles ecological patterns observed in various taxa, such as temperate birds and tropical trees (Fig 1B, 1C). This similarity is further supported by the fact that plasmid abundance distribution is well-fitted by classical species-abundance models, such as the Power-bend model (Fig 1A; see S1 Table for other models tested). Similar distribution patterns were also observed for the 3,277 and 1,168 plasmids within *Klebsiella* and *Salmonella* genus, respectively (S1A, S1B Fig), suggesting that these distributions are a general feature of plasmid assemblages across different bacterial hosts.

In ecology, distributions of species traits such as body size can summarize functional diversity and its role in enabling coexistence [64,65]. Like variability in dietary strategies in birds, or light-dependence strategies in trees, plasmid size distribution captures functional diversity (Fig 1D). Large plasmids are often conjugative, whereas smaller ones are typically nonconjugative [35–37]. Such diversity likely influences plasmid assemblages. Notably, size distributions are often multimodal, as exemplified by temperate birds, tropical trees and plasmids alike (Figs 1D, 1E and 1F; S1C, S1D for plasmid size distribution in *Klebsiella* and *Salmonella* [35,37,65–67]). This recurrent pattern suggests that for competing entities to coexist, they tend to be either strikingly similar or markedly different in their key traits [66,68,69].

While the mechanisms underlying RACs and trait-based distributions are intensively studied in ecology, they remain largely unexplored for plasmids within bacterial genomes.

### Applying biodiversity theories to plasmids

The similarities in diversity patterns between plasmids and ecological communities (Fig 1), support the idea that the conceptual frameworks of community ecology could be meaningfully applied to plasmid communities. A central paradox in ecology lies in the widespread coexistence of species despite competition for shared resources [75,76]. Such a situation should in theory lead to competitive exclusion, where only a few species persist. Over the past decades, fundamentally different theoretical frameworks have been proposed to resolve this paradox. Notably, ecological niche theory provides a deterministic view of biodiversity organization [77], while neutral theory emphasizes the role of stochastic processes [78]. More recent developments have successfully unified these perspectives within a single conceptual framework [66,68]. Considering that plasmids compete for their host as a common resource, we developed a modeling approach to illustrate how biodiversity theories can shed light on their eco-evolutionary dynamics and patterns of coexistence.

We built a minimal model combining two seminal frameworks: Stewart and Levin’s plasmid population dynamics [27] and the Lotka-Volterra competition model [79,80]. Despite its simplicity, this model captures key ecological principles and allows us to illustrate their relative contributions to plasmid assemblages. It examines the eco-evolutionary dynamics of plasmid species within a network of bacterial populations connected by dispersal, integrating plasmid innovation (the emergence of new species), heterogeneity in selection pressures, and stochastic processes—the latter generating demographic drift that can lead to plasmid species extinction. To systematically assess the weight of these mechanisms, we first present a deterministic version based on ecological niche theory, to show how competition between plasmids leads to exclusion unless trait differences allow their stable coexistence. We then introduce a second version, inspired by the neutral theory of biodiversity, to highlight the role of demographic stochasticity, dispersal, and innovation in shaping plasmid diversity. Finally, we integrate both perspectives into a unified model to illustrate how their interplay governs the structure of plasmid communities.

### Ecological niche theory for plasmids: Competitive exclusion and stable coexistence

Competition between species is a force of exclusion that shapes community assemblages. Classical ecological theory states that when multiple species compete for a single limiting resource supplied at a constant rate, coexistence is not possible [81–85]. Instead, the species capable of sustaining growth at the lowest level of resource availability will outcompete all others [86].

**Fig 2:**
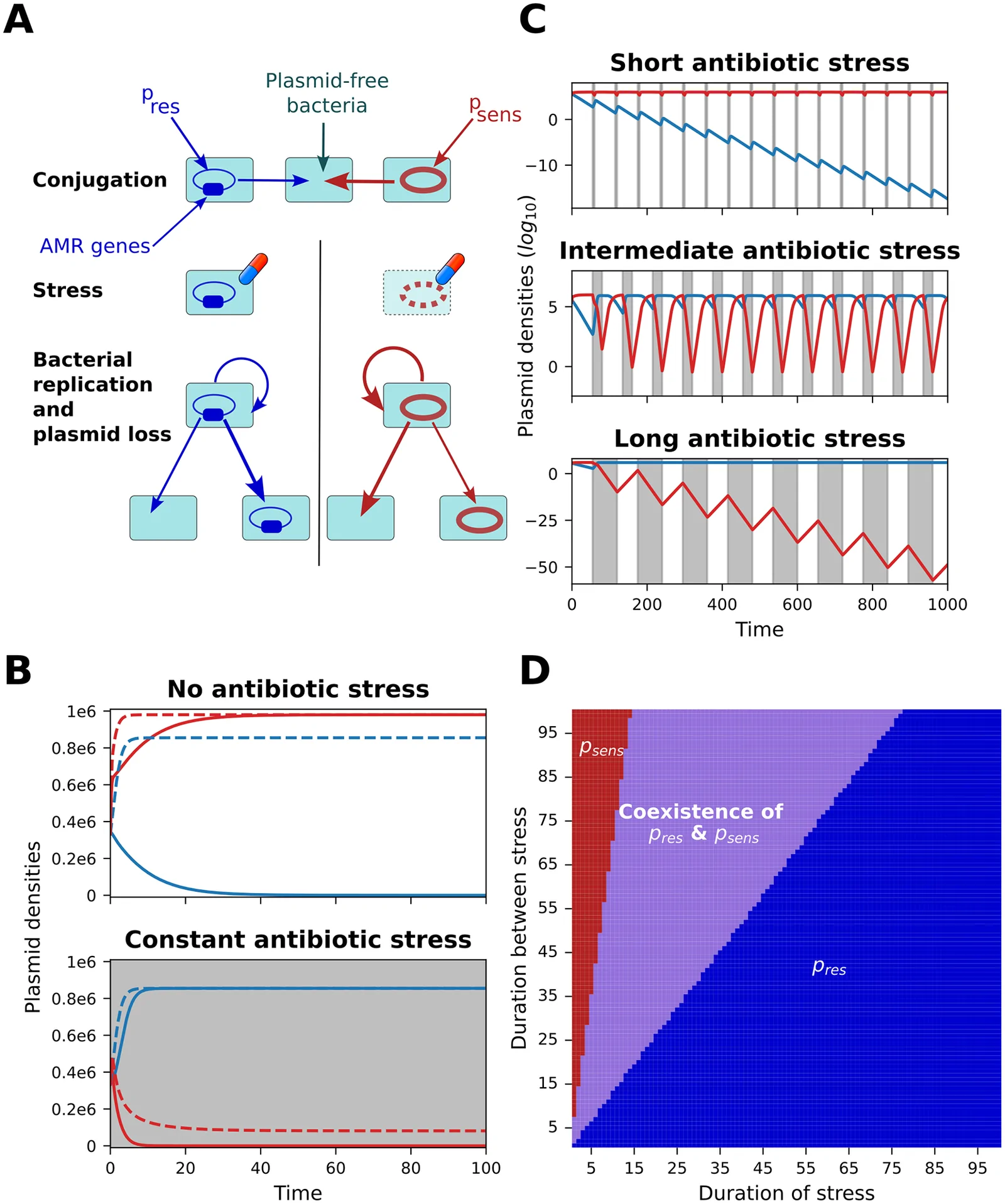
From exclusion to coexistence: how antibiotic stress shapes plasmid competition. Stress regimes flip the competitive balance between plasmids, sometimes creating room for coexistence. **(A)** A model with two strategies along two trade-off dimensions. $p_{res}$ (blue) transfers between bacteria at a moderate rate and carries an AMR gene. This offers strong resistance under stress at the expense of a slower host growth. $p_{sens}$ (red) spreads faster between hosts but carries no resistance, making it cheaper for the host yet vulnerable to stress. A second trade-off is defined: high horizontal transfer increases the probability that the plasmid is lost during cell division, making vertical transfer less reliable. **(B)** Under constant environments. Dashed lines show each plasmid alone (both can persist). Solid lines show them in competition. (Upper row) Without antibiotics (white background), the fast-spreading but sensitive plasmid wins. (Lower row) Under constant antibiotics (gray background), the resistant plasmid dominates. **(C, D)** Under fluctuating environments. **(C)** Antibiotic stress alternates between off (white zones) and on (gray zones). (Upper row) Short, rare pulses favor the sensitive plasmid. (Lower row) Long, frequent pulses enable the resistant plasmid to take over. (Center row) Intermediate pulses allow plasmid coexistence. **(D)** A map of all outcomes, from exclusion to coexistence, across stress fluctuation regimes. Parameters are listed in S2 Table. The data and code required to generate this Figure can be found in https://doi.org/10.5281/zenodo.21220021.

To illustrate this principle for plasmids, we consider two different plasmid species: (i) a resistant plasmid ($p_{res}$) that carries costly AMR genes with moderate HTR and (ii) a sensitive plasmid ($p_{sens}$) that lacks AMR genes but with a high HTR (Fig 2A and legend for details). Under the two antibiotic stress conditions illustrated, each plasmid species can grow when alone, i.e., spread through the bacterial population in the absence of competition from the other (dotted lines in Fig 2B).

As predicted by the competitive exclusion principle, only one of the two plasmids can persist under the constant environments tested. Without antibiotic stress, the sensitive plasmid dominates due to its efficient horizontal transfer, while the resistant plasmid is disadvantaged as it carries costly AMR genes and has a lower HTR (solid lines in Fig 2B). Conversely, under constant high antibiotic stress, the resistant plasmid prevails despite its lower HTR (solid lines in Fig 2C). Extending these simulations to multiple plasmid species with contrasted traits confirms that exclusion is generally the rule in constant environments. However, exclusion strongly slows down as the differences between the traits of plasmid species decrease (S2C, S2D Fig).

To resolve the paradox of coexistence of competing species, community ecology has proposed various stabilizing mechanisms that hinge on the criterion of invasibility. This principle posits that for species to coexist, each must be able to increase in frequency when rare, even in the presence of its abundant competitors [87,88]. We hypothesize that similar stabilizing mechanisms may operate in plasmid communities, maintaining their diversity despite intense competition for host resources.

Many stabilizing mechanisms rely on temporal or spatial fluctuations in resource availability, as they create opportunities for new ecological niches [87]. Such fluctuations should be relevant for plasmid coexistence because their bacterial hosts frequently experience variable environmental conditions, both in space and time [89]. Here, we focus on periodic antibiotic stress.

When the two plasmids—both capable of spreading under all considered stress regimes when alone—compete under fluctuating antibiotic exposure, stress duration and frequency determine the outcome of competition (S2A Fig). Short, rare stress pulses favor the sensitive plasmid, which outcompetes the resistant one because exposure to stress is too rare for the resistant plasmid to grow sufficiently (Fig 2D and upper row in Fig 2C). On the contrary, long, frequent stress pulses favor the resistant plasmid (Fig 2D and lower row in Fig 2C). Coexistence occurs under a wide range of intermediate regimes, where both plasmids are able to grow sufficiently when rare during their respective favorable conditions (Fig 2D and center row in Fig 2C).

Thus, niche theory provides a deterministic framework to understand plasmid dynamics and their potential for coexistence despite the inherently exclusionary nature of competition. In addition to mechanisms based on spatio-temporal environmental heterogeneity, other stabilizing processes may also contribute to the maintenance of plasmid diversity (see Discussion).

### Neutral theory for plasmids: Ecological drift, dispersal and emergence of new variants

Unlike niche partitioning theory, which explains biodiversity through differences in species traits, the neutral theory of biodiversity assumes that purely stochastic processes can account for key aspects of community organization [78,90–93]. Neutral models consider an extreme scenario in which all species are functionally equivalent, sharing identical demographic parameters [78,94]. Despite lacking niche-based stabilizing processes, neutral models can transiently generate species-rich communities that reproduce key biodiversity patterns such as RACs (Fig 1) [78,95,96], which has challenged traditional deterministic interpretations of biodiversity organization. Neutral theory relies on three key principles: (i) competitive exclusion is slow when species are similar; (ii) exclusion is further slowed down when communities are connected by dispersal; and (iii) the inevitable erosion of diversity can be counterbalanced by rare events such as immigration or speciation.

**Fig 3:**
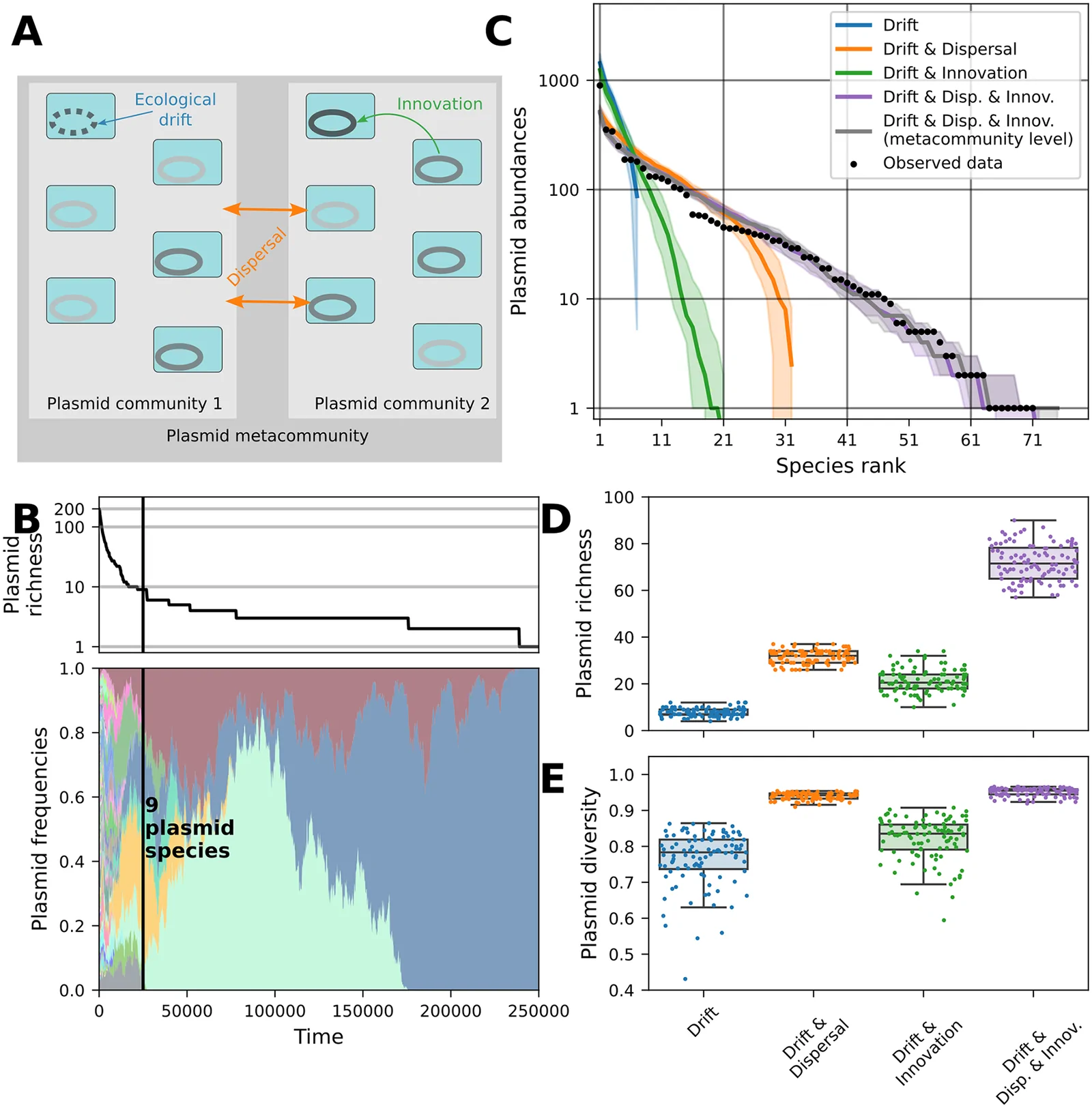
When chance, dispersal among bacterial populations, and innovation sustain plasmid diversity. **(A)** A neutral playground for plasmid. We model a plasmid metacommunity made of four bacterial populations, each hosting a community of plasmids. These communities exchange plasmids through bacterial dispersal. All starts with 200 plasmid species, identical in traits and demographic parameters (S3 Table). Over time, stochastic stress creates bottlenecks which amplify demographic stochasticity. Innovation allows new plasmid species to emerge, inheriting traits from their ancestors. This setup lets us explore how demographic stochasticity, dispersal, and innovation shape plasmid diversity. **(B)** Ecological drift in action. Without dispersal or innovation, stochasticity alone shapes diversity. (Upper row) Plasmid species richness declines over time as species go extinct. (Lower row) Frequencies of plasmid species fluctuate stochastically. After the early loss of many species, reaching complete exclusion (one species remaining) can take a long time. **(C)** Simulated neutral plasmid diversity versus real data. Observed plasmid diversity (black dots) is compared with simulations under four scenarios: (i) drift only (blue); (ii) drift and dispersal (orange); (iii) drift and innovation (green); and (iv) drift and dispersal and innovation (purple). The gray curve shows diversity at the meta-community scale with dispersal and innovation combined. For each case, the median (curve) and interquartile range (area) are represented from 25 model simulations. When all stochastic processes are included, the resulting plasmid abundance distributions resemble those observed in *E. coli* genomes: a few dominant species, many intermediate ones, and a long tail of rare species. **(D)** Maintenance of local richness. Effects of dispersal and innovation do not just add up; they reinforce each other. Together, they boost plasmid richness far beyond what either could achieve alone. **(E)** Maintenance of local heterogeneity. Gini–Simpson index values (measuring the heterogeneity of community structure based on the species relative abundance, representing the probability that two randomly sampled plasmids belong to different species, see Materials and methods) reveal that dispersal is the main driver of within-community plasmid diversity. Dispersal counteracts local extinctions by leveraging compositional differences among communities. For panels **D** and **E**, ANOVA followed by Tukey post-hoc tests revealed significant differences for all global and pairwise comparisons (*p* < 0.001), with the exception of plasmid heterogeneity between the ‘drift and dispersal’ and ‘drift and dispersal and innovation’ scenarios in panel **E**. The data and code required to generate this Figure can be found in https://doi.org/10.5281/zenodo.21220021.

To test whether these key principles can explain plasmid diversity, we built on our previous model to develop a neutral metacommunity model as follows: (i) we incorporate demographic stochasticity at multiple levels through random sampling affecting bacteria and plasmid dynamics; (ii) all plasmid species have identical traits and equivalent demographic parameters; (iii) we model multiple plasmid communities connected by bacteria-mediated plasmid dispersal; and (iv) new plasmid variants emerge stochastically bearing the same traits as the ancestral plasmids (Fig 3A).

We first illustrate ecological drift by following the fate of plasmid species in a single community without innovation (Fig 3B). Richness rapidly declines from 200 to about 100 species within a thousand generations, followed by slower extinctions. Plasmid relative abundances shift quickly but competitive exclusion of all species except one can be very long (230.000 generations in Fig 3B). At intermediate times (e.g., 25,000 generations), only a handful of species remain, a pattern confirmed across 25 replicate simulations (blue points in Fig 3D). The resulting RACs show a few dominant species and others with moderate abundances, but no very rare ones (blue line in Fig 3C).

Introducing bacterial-mediated plasmid dispersal between communities slows down exclusion, maintaining more species and higher heterogeneity within each community (Fig 3D, 3E), but still produces RACs with few rare species (orange line, Fig 3C). The innovation process (speciation or new variant formation) may be considered a major phenomenon in plasmids [97]. It can arise not only through mutation or immigration from outside the plasmid metacommunity, but also via plasmid recombination or through the gain or loss of transposons [98]. Such innovation can counterbalance plasmid extinctions, stabilizing plasmid diversity and leading to patterns with several extremely rare species (green line in Fig 3C and 3D, 3E). Importantly, these results remain qualitatively similar when alternative plasmid traits are considered, particularly under conditions combining lower HTRs with highly efficient vertical transmission (S4 Fig).

When drift, dispersal and speciation act together, simulated plasmid abundances distributions closely match empirical data at both community and metacommunity levels (see purple and gray lines in Fig 3C, respectively). The model successfully fits the observed data by inferring both the innovation and the dispersal rates through the exploration of the parameter space (S3A Fig). Under these conditions, plasmid richness remains high (Fig 3D): innovation continuously generates many rare plasmid species, while a few ones become very abundant. Crucially, a substantial number of plasmid species are maintained at intermediate abundances through dispersal. This interplay generates high local plasmid richness and significant heterogeneity within each community (Fig 3D, 3E).

More generally, within this neutral framework, both local and global plasmid richness (number of species; S3B Fig) and diversity (here quantified by the Gini-Simpson Index, i.e., the probability that two randomly chosen plasmids belong to different species; S3C Fig) increase with both innovation and dispersal rates.

Neutral theory, when applied to plasmid dynamics, can therefore recreate a substantial part of their community structure. As in ecology, it could provide powerful tools to generate null hypotheses aimed at testing the role of trait differentiation in shaping plasmid assemblages [92,99,100]. One way to assess the influence of trait differentiation through modeling is to assume that innovation does not simply produce new species similar to ancestral ones, but species with distinct traits. Such trait differences might theoretically either accelerate competitive exclusion or, conversely, promote stable coexistence.

### Unification of biodiversity theories applied to plasmids

Following the neutral theory’s challenge to the deterministic niche theory perspective, several studies have proposed a synthesis combining the two frameworks [66,68,101–104]. Building on this, we asked: is plasmid diversity predicted by neutrality robust to trait differentiation? Can the evolution of plasmid strategies both drive exclusion and promote coexistence?

**Fig 4:**
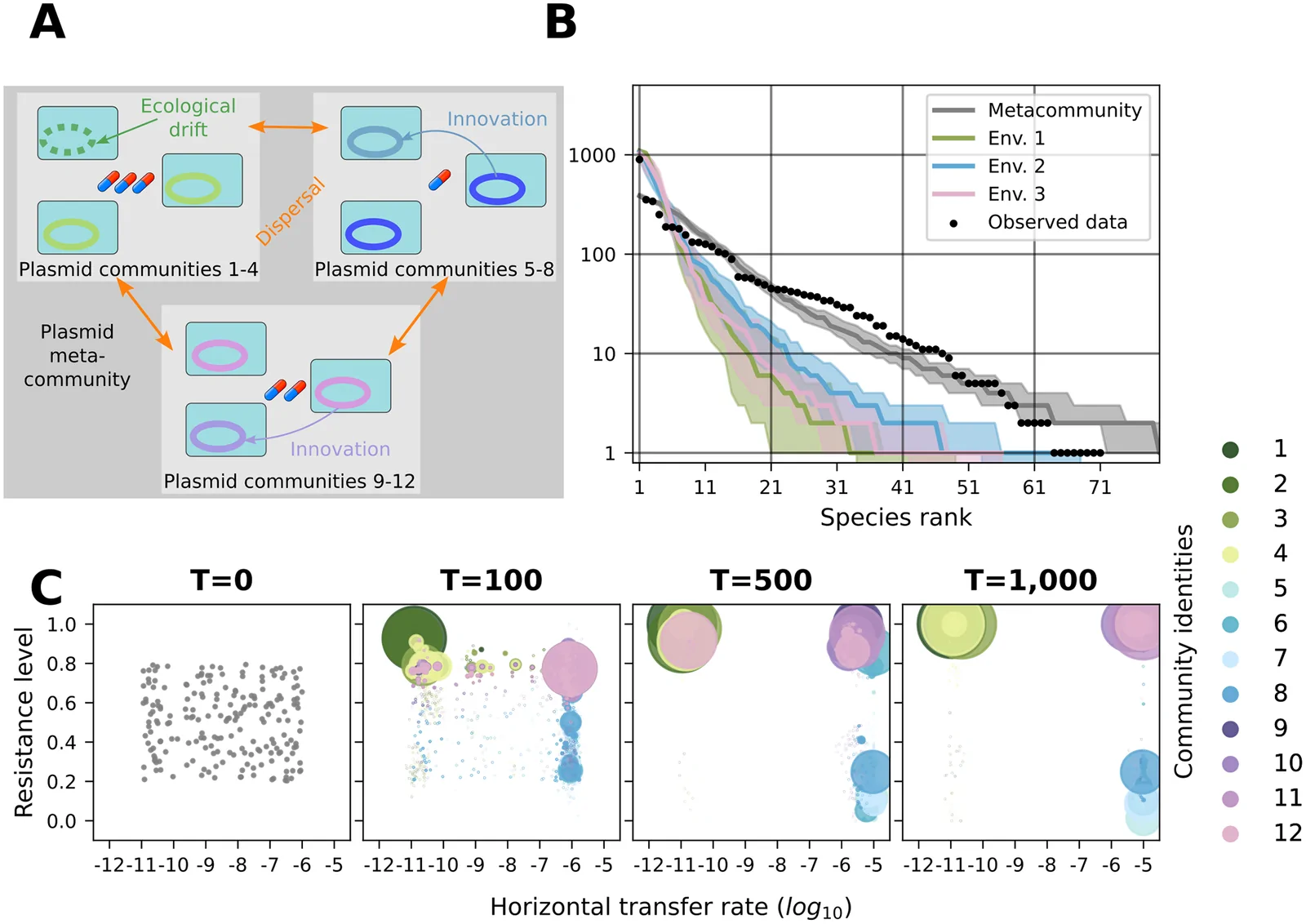
A unified view of plasmid biodiversity, from neutral drift to adaptive clustering. **(A)** How the model works. We simulate a plasmid metacommunity of 12 local communities connected by bacterial-mediated plasmid dispersal. Each local community faces a different environment, defined by antibiotic stress regime: four with long, frequent stress (in greens, environment 1); four with short, rare stress (in blues, environment 2); four with intermediate stress (in purples, environment 3) (Parameters are listed in S4 Table). Initially, all communities start with the same set of 200 plasmid species, whose horizontal transfer rates (HTR) and resistance levels (RL) are randomly drawn (see Materials and methods). Over time, plasmids can “innovate” creating new species that inherit parental traits with slight modification (see Materials and methods). **(B)** Model meets observed plasmid abundance distribution. At the metacommunity level, the simulated RACs (in gray) are qualitatively similar to the patterns observed in *E. coli*: a few dominant plasmid species, many with intermediate abundances, and a long tail of rare species. At the local scale, plasmid richness significantly differs across environmental regimes (ANOVA *p*-value<0.05). Specifically, environment 1 (chronic stress) is characterized by a lower richness than environment 2 (low stress) (Tukey post-hoc test *p*-value<0.01). Solid lines show the median rank-abundance calculated from 25 simulations, with shaded areas indicating the interquartile variation. **(C)** Eco-evolutionary sorting in action. Each dot represents a plasmid species in trait space (HTR on the x-axis, RL on the y-axis). Dot size reflects abundance; color indicates the community identity. (T = 0) Initial species traits are randomly distributed with equal abundances. (T = 100) After 100 generations, plasmids begin diverging toward peripheral strategies, high/low resistance, high/low transfer, or both. (T = 500 and T = 1,000) After 500 and 1,000 generations, three distinct clusters have emerged: (i) high RL with low HTR, (ii) high HTR with low RL, and (iii) high HTR with high RL. This reflects an eco-evolutionary sorting driven by local stress regimes and connectivity across communities. The data and code required to generate this Figure can be found in https://doi.org/10.5281/zenodo.21220021.

To address this, we developed a unified model incorporating the four main processes likely to shape the eco-evolutionary dynamics of plasmids: ecological drift, dispersal, speciation, and trait differentiation. Building upon the neutral baseline, the model introduces three key features: (i) environmental heterogeneity: local plasmid communities experience distinct antibiotic stress regimes. We selected three highly contrasted regimes to capture a broad range of ecological contexts: (a) environments under intense selective pressure (long and frequent exposure), (b) environments with minimal exposure (short and low frequency pulses), and (c) intermediate regimes; (ii) initial trait variation: plasmids start with random values of HTR and resistance level (RL) (Fig 4C); (iii) trait evolution: new plasmid variants arise with slightly modified traits inherited from their ancestor (Fig 4A).

Early phases of plasmid assembly combine rapid diversity loss with increasing trait differentiation (time-lapse video S1 Movie). During the first hundred generations, plasmid richness declines sharply, while traits diverge (Fig 4C). Later, overall richness stabilizes, but community composition continues to shift through innovation and dispersal (Fig 4C).

Over time (by ~1,000 generations), plasmids sort into three adaptive clusters shaped by stress regimes (Fig 4C). The first cluster (low HTR, high RL) is similar to multi-resistant, mobilizable plasmids that dominate under frequent and prolonged antibiotic stress. Long-term richness remains stable (S5B1 Fig), while functional diversity can fluctuate on short timescales due to incoming plasmids with high HTR (S5B3 Fig). The second cluster (high HTR, low RL) is akin to conjugative plasmids without resistance genes, acting mainly as parasites and prevailing in communities with rare and short stress pulses. Dispersal may introduce resistant plasmids, causing brief increases in functional diversity (time-lapse video S1 Movie). The last cluster (high HTR, high RL) resembles conjugative and resistant plasmids, thriving in intermediate, fluctuating environments, where both richness and functional diversity remain relatively stable despite environmental fluctuations. Notably, the emergence of these functional clusters provides a mechanistic explanation for the multimodal plasmid size distributions observed empirically (Fig 1D), as plasmid size is intrinsically linked to the genetic cargo required for mobility and resistance [35,36]. Furthermore, we confirmed the robustness of these evolutionary dynamics by running simulations where the plasmid HTR was allowed to evolve across a broader range (1e−15 to 1e−5) under a highly efficient vertical transmission ($\varepsilon_{min}=\text{0}.\text{9}\text{9}\text{9}$ and $\varepsilon_{max}=\text{0}.\text{9}\text{9}\text{9}\text{9}\text{9}\text{9}$, i.e., a plasmid loss rate between 1e−3 and 1e−6). Under these stringent parameter conditions, we consistently observed the emergence of divergent plasmid clusters in the trait space, maintaining a robust fit with the empirical RACs (S6 Fig).

Although divergent trait evolution generates three clusters, plasmids with similar traits coexist within each cluster over long periods. This occurs because competitive exclusion is slow and continuously offset by the emergence of new variants. This pattern aligns with the concept of emergent neutrality [66,68,69] suggesting that species (or plasmids) can coexist either by being sufficiently different (stabilization mechanisms between clusters) or by being sufficiently similar within a cluster (extinction slowed down by dispersal and counterbalanced by innovation). Our unified model predicts plasmid abundance distributions consistent with empirical data when dispersal and innovation rates are inferred through an extensive exploration of the parameter space (Figs 4B and S5A, see Materials and methods). Interestingly, the resulting RACs vary significantly depending on the environmental fluctuation regimes within each locality (Fig 4B). Specifically, plasmid richness is highest in environments where stress is rare (blue line in Fig 4B); it is intermediate in environments with highly fluctuating exposure (purple line in Fig 4B), and lowest in environments under very frequent stress (green line in Fig 4B). This occurs because intense antibiotic pressure accelerates competitive exclusion relative to stress-free conditions (see S2C, S2D Fig), thereby reducing local species diversity.

Sensitivity analyses (S5 Fig) reveal that global richness (number of species), diversity (probability that two randomly sampled plasmids belong to different species), and functional diversity (capturing the variance in HTR and RL traits) all increase with the innovation rate. However, global diversity decreases as dispersal increases (S5 Fig). High dispersal rates erode the structuring effects of spatial heterogeneity [105], which otherwise favor the coexistence of distinct clusters. By homogenizing the metacommunity, high dispersal allows a subset of dominant species to outcompete others globally. Conversely, because multiple species may possess highly similar traits, dispersal can facilitate local coexistence of similar species, slightly increasing local diversity without significantly affecting local functional diversity (S5 Fig).

Ultimately, integrating deterministic processes (traits differences) with stochastic ones (drift, dispersal, and innovation) thus offers a powerful framework to predict how plasmid diversity unfolds across contexts—from clinical settings to natural microbiomes with minimal human impact.

## Discussion

Genomics analyses revealed the huge diversity of plasmids [106]. While their molecular biology is becoming increasingly well characterized, the forces sustaining this diversity remain elusive. We show that ecological theories offer a powerful framework for understanding plasmid assembly and eco-evolutionary dynamics. This proposal is inspired by the analogies between species diversity within ecological communities and plasmid diversity within bacterial hosts (Fig 1). These parallels are reinforced by our model, which demonstrates that competition acts as a primary structuring force. Our study helps lay the foundations for a “plasmid ecology” by showing how selection, drift, dispersal, and innovation [104] jointly shape these plasmid communities (Fig 4).

### Neutrality as a baseline

Neutral models replicate a hallmark of plasmid assemblages: a few dominant species coexisting with a “long tail” of rare ones (Fig 3). This suggests that such patterns do not strictly require functional trait differences. This echoes the paradigm shift triggered by neutral theory in ecology, which challenged deterministic views by demonstrating that dramatic, stable differences in species-abundance and complex spatial assemblages can emerge without invoking niche differentiation [78]. In our model, the slow competitive exclusion of similar plasmids is necessary but insufficient to produce realistic RACs. Instead, these distributions emerge when competitive exclusion is significantly delayed by bacterial-mediated dispersal, and further counterbalanced by innovation—which is expected to occur frequently in plasmids through mutation or recombination.

Although not explicitly explored in the present study, the neutral theory of plasmid diversity could be significantly extended. Specifically, the concept of plasmid “dispersal” should encompass horizontal transfer across species boundaries. In this view, plasmid communities are interconnected not only by spatial movement between host populations (e.g., such as connectivity between distinct microbiotas) but also by local cross-species transfer (e.g., within the microbiota). This multi-level connectivity creates a “meta-metacommunity of plasmids” which could further decelerate competitive exclusion and reinforce structured patterns without necessitating trait-based selection. Much like in classical ecology, this neutral framework provides a robust null model to test how plasmid trait-based selection emerges as a dominant structuring force of pangenomes [92,99,100].

### Toward a unified theory of plasmid diversity.

Despite the relevance of neutral processes, plasmid dynamics and assemblages should also depend on vast differences in their functional traits such as mobility, copy number, segregation systems and accessory genes (e.g., resistance, virulence). The magnitude of these trait differences could strongly determine the relative performance of plasmids (fitness differences), and the speed of competitive exclusion [87,107,108]. This is illustrated by the fact that in a constant environment, competitive exclusion is accelerated as trait differences increase along a single niche axis (S2C, S2D Fig).

Conversely, specific trait differences can lead to ecological niche partitioning and stabilize coexistence, particularly when integrating a broader range of niche axes or more complex competitive contexts—such as temporally or spatially fluctuating environments and diverse host species. This stabilization requires satisfying the key criterion of mutual invasibility: each variant must possess distinct traits (niche partitioning) which provide a fitness advantage when rare, even when its competitors are abundant.

By combining the delayed competitive exclusion of highly similar plasmids with the stabilization provided by niche partitioning, our findings align with the theory of emergent neutrality, which reconciles niche and neutral perspectives [66,68]. In that sense, our model shows that spatio-temporal variation in stress exposure drives the emergence of distinct, coexisting plasmid clusters, each specialized for a specific stress regime (Fig 4C). For instance, environments characterized by extremely rare stress pulses favor ‘parasitic’ plasmids (low resistance, high HTR) that rely on horizontal persistence. In contrast, intermediate fluctuating environments promote plasmids that combine high horizontal transfer with high RLs. Finally, chronic stress favors plasmids with high vertical transmission (low HTR) and high resistance. This latter group mirrors plasmids that have minimized their fitness cost, for example through compensatory mutations [109]. If, however, the plasmid remains significantly costlier under such persistent pressure, these conditions might ultimately favor the chromosomal capture of resistance genes, thereby eliminating the metabolic burden of plasmid maintenance while preserving the adaptive trait [110].

Our analysis of plasmid diversity across heterogeneous environments also reveals that intense antibiotic pressure does not merely favor the emergence of specific plasmids; it also accelerates competitive exclusion, thereby reducing local species richness (Figs 4B and S2). This finding may help clarify the observed shifts in plasmid diversity between the pre-antibiotic era and the modern clinical landscape [111]: while the widespread use of antibiotics has favored the emergence of conspicuously large and complex modern plasmids, often through fusion and multi-drug resistance acquisition, it likely altered the composition of the broader ‘long tail’ of rare plasmids. However, our results also indicate that these diversity patterns should vary significantly depending on local selective pressures (e.g., low versus high antibiotic exposition) and the degree of connectivity between contrasted environments. This prediction remains to be further investigated in contemporary plasmid communities.

More broadly, plasmid coexistence should be driven by complementary processes: plasmids can coexist either by being sufficiently different (between clusters) through niche partitioning, or by being sufficiently similar (within a cluster), where “unstable” coexistence is sustained by extremely slow competitive exclusion. This dual mechanism explains how plasmid communities can maintain both high taxonomic richness and distinct functional signatures across a wide range of ecological settings, from natural microbiomes to clinical environments.

### Stabilization of coexistence

Beyond spatio-temporal environmental fluctuations modeled in this study, niche partitioning can emerge through resource partitioning or apparent competition [112].

#### Host species partitioning and feedback loops.

Coexistence stabilization can occur through resource partitioning, where species exploit distinct resources through different trade-offs [86]. The most direct translation to plasmids is host species partitioning (variation in host range). Plasmid may specialize on specific bacterial hosts within a shared pool or adopt generalist strategy with lower efficiency in any single host [10]. Under this mechanism, plasmid performance is indirectly tied to the host community structure. This necessitates a “plasmid ecology” intimately linked to bacterial community ecology: by influencing host traits (resistance, immunity, virulence), plasmids participate in partitioning host niches and structuring bacterial communities. In turn, this structure determines horizontal transfer opportunities, creating a feedback loop between the dynamics of bacteria and plasmid meta-community. Interestingly, epidemiology, long focused on the one-host-one-pathogen relationship, has recently drawn from meta-community theory to formalize pathogen-host diversity [113,114], providing inspiration for developing a coupled plasmid-bacteria ecology.

#### Apparent competition and immunity.

Coexistence stabilization may also involve apparent competition, where growth is limited by shared natural enemies rather than resources [87,115,116]. In plasmids, this translates to escaping host’s immune defenses (e.g., CRISPR-Cas or restriction-modification systems) [117]. Coexistence could be stabilized through trade-offs: plasmids with lower intrinsic horizontal transfer efficiency might be more competitive in evading bacterial immunity, and vice versa.

### Toward a general ecology of MGEs

Although our framework focuses on plasmids, its principles could be extended to other MGEs, including phages, transposons, and genomic islands. Phages may compete for bacterial hosts and display a wide array of traits and interaction modes [17,118], while transposons compete for genomic insertion sites [119,120]. A general ecology of MGEs must also account for nested biological hierarchies, such as transposons parasitizing plasmids, and recognize that the processes structuring one level of MGEs inevitably impact the others.

### Future directions: Bridging disciplinary gaps

A new ecology of MGEs is emerging, demanding deep integration across theoretical ecology, dynamical modeling, microbial ecology and genomics, and empirical approaches. While ecology brings powerful tools to study eco-evolutionary dynamics and species assemblage, “MGE ecological theory” must embrace its unique biological features: high innovation rates, horizontal transfers, and complex interactions with their host and among MGEs. Microbial genomics, in turn, offers an unparalleled test bed for biodiversity theory, especially when combined with ecologically informed sampling and high-resolution sequencing, from cells to metacommunities. These insights must be grounded in experiments that parameterize models and validate predictions. This integration is not just conceptual: it is critical for confronting real-world challenges, including the global spread of antibiotic resistances.

## Materials and methods

### Diversity patterns

#### Plasmid diversity.

To mitigate the risk of biased sampling and the over-representation of pandemic clones commonly found in public databases, we implemented a stringent genomic de-replication step. We filtered all complete *E. Coli, Klebsiella,* and *Salmonella* genomes available on NCBI RefSeq (last accessed March 2023) using a sequence-based approach to exclude closely related strains. By applying a MASH distance threshold of <0.0001, we ensured that our dataset consists of nonredundant, genomically independent hosts, thereby avoiding the inclusion of pseudo-replicates from clonal outbreaks. From this de-replicated set, we extracted a total of 6,131 (*E. Coli*), 5,149 (*Klebsiella*) and 1,517 (*Salmonella*) plasmids for downstream analysis. We then clustered these plasmids in plasmid taxonomic units or PTU using COPLA v1.0 and default parameters [62]. We obtained 71 (*E. Coli*), 61 (*Klebsiella*) and 47 (*Salmonella*) PTU containing a total of 4,411 (*E. Coli*), 3,277 (*Klebsiella*), and 1,168 (*Salmonella*) plasmids. The remaining plasmids were not assigned to known PTUs. We then could count the number of plasmids for each PTU. Data available in PTU_count.tsv (and plasmid_data_sup.xlsx for *Klebsiella* and *Salmonella* genera).

We also extracted the size (in base pairs) of all the clustered plasmids, which we used to calculate the average plasmid size for each of the PTUs. Data available in Plasmid_size_mobility_PTU_coli.tsv (and plasmid_data_sup.xlsx for *Klebsiella* and *Salmonella* genera).

Then we plot a histogram of mean PTU size (on a log10 scale) as well as the Gaussian Mixture Model.

#### Bird diversity.

We retrieved bird species occurrences on the Global Biodiversity Information Facility database (gbif.org) with the following filters:Scientific name = Animalia, Chordata, Aves

Basis of record = Human observation

Dataset = iNaturalist Research-grade Observations

Year = 2024 (Between start of 2024 and end of 2024)

Administrative areas = USA.51_1 (Wyoming, USA)

These data are given in the birds_wyoming.csv file.

Citation: GBIF.org (19 June 2025) GBIF Occurrence

Download https://doi.org/10.15468/dl.rvf9ry

We retrieved bird body sizes from Zheng et al’s paper [71] and stored them in the file bird_data.csv. Using these data, we calculate the mean size of each bird species. Then we plot a histogram of the mean size of each bird species (on a log10 scale) as well as the Gaussian Mixture Model.

#### Tree diversity.

To illustrate tropical forest tree diversity, we choose to focus on a single community, the widely studied Barro Colorado Island (BCI) in Panama. First, for the SAD representation, we retrieved tree species-abundances from the BCI dataset in the vegan package on R which we converted as a csv file (BCI.csv) to export in python. Second, for the tree height diversity, we used the data from Wright and colleagues [72], which for the same tree community calculated the mean height of each tree species based on its six highest individuals. These data are given in the BCI_traits.txt file. Then we plot a histogram of mean tree species height (on a log10 scale) as well as the Gaussian Mixture Model.

#### Fitting RAC.

To fit the observed RAC, we use the *fitsad* function from the R package *sads* (version 0.6.5) integrated into our in Python workflow. We tested six classical species-abundance models for RAC (namely Broken-stick, Fisher’s log-series, Geometric, Poisson-lognormal, Lognormal and Pueyo’s power-bend). Model selection was based on the Akaike Information Criterion (AIC) with the lowest value of AIC identifying the best-fit model. Once the best model is found, we generated 500 independent random draws from the theoretical distribution to calculate and represent the 95% confidence intervals (CI).

#### Determining the number of modalities in traits.

To determine the number of modalities in trait distributions, we used Gaussian Mixture Models (*GaussianMixture* function from *sklearn.mixture* Python package). For each trait, we evaluated models consisting of a mixture of 1–7 gaussian components. We used Bayesian Information Criterion (BIC) to compare the different models, selecting the optimal number of components based on the lowest BIC value.

### Modeling plasmid eco-evolutionary dynamics

To model plasmid eco-evolutionary dynamics within a bacterial (meta)population, we combine two seminal approaches: Stewart and Levin’s plasmid dynamics [27] and the Lotka-Volterra competition model [79,80]. We obtain a population-centered compartmental model in which plasmids compete for transfer between bacterial hosts.

We developed three versions of the model tailored to different objectives: (i) a deterministic model without plasmid evolution; (ii) a stochastic model allowing plasmid innovation (new species can emerge) but without trait variation; and (iii) a stochastic model in which plasmid traits vary and thus evolve over time.

#### Deterministic model.

We first build a deterministic, continuous time model. Let $B_{\text{0}}$ denote the density of plasmid-free bacteria and $B_{i}$ for i=1,...,n the densities of bacteria carrying plasmid i. Plasmid i conjugation is modeled as the transfer of a plasmid copy to a plasmid-free bacteria, occurring at a rate given by the mass action law $\alpha_{i}B_{\text{0}}B_{i}$, where $\alpha_{i}$ capture the plasmid’s transfer ability.

The per-capita growth rate of plasmid -carrying bacteria is given by: (i) an intrinsic bacterial replication rate $r_{i}\varepsilon_{i}$ where $r_{i}$ accounts for the plasmid cost and $\varepsilon_{i}$ is the probability that the plasmid is effectively transmitted to the daughter cell during replication, (ii) minus an antibiotic stress-induced mortality rate $I(t)\left(\text{1}-s_{i}\right)$, where I(t) is a time-dependent antibiotic stress induced mortality rate and $s_{i}$ the level of plasmid antibiotic resistance; and (iii) minus a density-dependence competition with all bacteria, at rate dB, where d is a per capita competition rate and $B=\sum_{i=\text{0}}^{n}B_{i}$ the total bacterial density, regardless of plasmid composition.

Plasmid-free bacteria grow similarly, with intrinsic rate $r_{0}$, antibiotic mortality (no resistance) I(t), and density-dependent competition dB.

Finally, plasmids can be lost during bacterial replication, generating plasmid-free bacteria at a total rate $\sum_{i=1}^{n}\;r_{i}(1-\varepsilon_{i})\;B_{i}$. All the variables and parameters are summarized in S2 Table. Put together, the dynamics of the bacterial population densities are thus modeled as follows:


$$\begin{array}{c} B_{\text{0}}^{\prime }(t)=\left(r_{\text{0}}-I(t)-dB(t)\right)B_{\text{0}}(t)-B_{\text{0}}(t)\sum_{i=\text{1}}^{n}\alpha_{i}B_{i}(t)+\sum_{i=\text{1}}^{n}r_{i}\left(\text{1}-\varepsilon_{i}\right)B_{i}(t) \end{array}$$ (1)

$$\begin{array}{c} B_{i}^{\prime }(t)=\left(r_{i}\varepsilon_{i}-I(t)\left(\text{1}-s_{i}\right)-dB(t)\right)B_{i}(t)+\alpha_{i}B_{\text{0}}(t)B_{i}(t)\;\;for\;\;i=\text{1},...,n \end{array}$$

Two trade-offs between plasmid traits guide model parametrization. First, carrying AMR genes (represented by the RL $s_{i}$ in our model), imposes a cost that reduces the replication rate $r_{i}$ of plasmid-carrying bacteria:


$$\begin{array}{c} r_{i}=r_{max}-\left(r_{max}-r_{min}\right)s_{i} \end{array}$$ (2)

Second, the ability of horizontal transfer can result in an increase in loss probability during bacterial replication. Considering that plasmid HTRs span several orders of magnitude [38,41], we consider that the cost of this HTR trait is expressed on the same ${\text{log}}_{\text{1}\text{0}}$ scale for the vertical transmission efficiency trait. We thus, introduce a trade-off between the min-max normalized HTR $\alpha_{i}^{\prime }$ and the vertical transmission efficiency $\varepsilon_{i}$:


$$\begin{array}{c} \varepsilon_{i}=\varepsilon_{max}-\left(\varepsilon_{max}-\varepsilon_{min}\right)\frac{\alpha_{i}^{\prime }}{\alpha_{max}} \end{array}$$ (3)

where $\alpha_{i}^{\prime }=\frac{{\text{log}}_{\text{1}\text{0}}\left(\alpha_{i}\right)-{\text{log}}_{\text{1}\text{0}}\left(\alpha_{min}\right)}{{\text{log}}_{\text{1}\text{0}}\left(\alpha_{max}\right)-{\text{log}}_{\text{1}\text{0}}\left(\alpha_{min}\right)}$ with$\alpha_{min}$ and $\alpha_{max}$ being the minimal and maximal HTR respectively. In the simulations, we always take $\alpha_{min}=\text{1}\text{e}-\text{1}\text{2}$ and $\alpha_{max}=\text{1}\text{e}-\text{5}$.

#### Stochastic model.

We build a stochastic, discrete time version of the model defined by [1]. Unlike the previous deterministic model, this version includes plasmid innovation, allowing new plasmid species to emerge from a single mutated plasmid at a constant innovation rate τ. In addition, this version models interconnected bacterial populations, a *bacterial metapopulation*, corresponding to a *plasmid metacommunity* made of local communities. Bacteria disperse between populations at a constant rate Γ.

Initial bacterial densities $B_{i}(\text{0})\;for\;i=\text{0},...,n$are taken as $B_{\text{0}}(\text{0})=\text{1}\text{0}\text{0}\text{0}$ and $B_{i}(\text{0})=\text{1}\text{0},\forall i>\text{0}$, i.e., with equal initial density of all plasmids and two-orders-of-magnitude more plasmid free bacteria. Stress intensity time series I(t) for all local communities are pre-generated as follows: stress and no stress durations were drawn from exponential distributions with means $d_{\text{1}}$ and $d_{\text{2}}$ respectively. Stress intensity was kept constant during each stress episode, while set to zero during no stress periods. The process was iterated until the total simulation time T (with time step $\Delta_{t}$) was reached.

In each community, population densities are updated iteratively in four steps. (i) **Dispersal**: the number of bacteria dispersing from a community $c_{\text{1}}$ to another community $c_{\text{2}}$ is drawn from a binomial distribution, $D_{c_{\text{1}}\to c_{\text{2}}}\sim Bin\left(B_{.,c_{\text{1}}},\Gamma \Delta_{t}\right)$ and vice versa. (ii) **Replication**: the number of replicating bacteria is drawn as $G_{i}\sim Bin\left(B_{i}(t),r_{i}\Delta_{t}\right)$. For plasmids-carrying bacteria, replicating ones are partitioned into those that keep their plasmids $R_{i}\sim Bin\left(G_{i},\varepsilon_{i}\right)$ and those that lose it, $\overset{―}{R_{i}}=G_{i}-R_{i}$. (iii) **Mortality**: the number of dead bacteria is drawn as $L_{i}\sim Bin\left(B_{i}(t),\left(dB(t)+I(t)\left(\text{1}-s_{i}\right)\right)\Delta_{t}\right)$ where B(t) is the total density of bacteria in the local community. (iv) **Horizontal transfer and innovation:** the number of newly infected plasmid-i-carrying bacteria is drawn as $A_{i}\sim Bin\left(B_{\text{0}}(t),\alpha_{i}B_{i}(t)\Delta_{t}\right)$ and the number of innovating plasmids $N_{i}\sim Bin\left(B_{i}(t),\tau \Delta_{t}\right)$. The total number of plasmid species is updated as $n^{\prime }=n+\sum_{i=\text{1}}^{n}N_{i}$. In the neutral model, new plasmid species inherit the exact same traits of their ancestor, thus $\alpha_{j}=\alpha_{i},s_{j}=s_{i},r_{j}=r_{i},\epsilon_{j}=\epsilon_{i},\text{ for }j=1,...,N_{i}$. On the contrary, in the unified model, new plasmid traits are randomly drawn from a truncated normal distribution N as follows: $a_{j}=min\left(max\left(\text{1}\text{e}-N\left({\text{log}}_{\text{1}\text{0}}\left(\alpha_{i}\right),\text{0}.\text{1}\right),\text{1}\text{e}-\text{1}\text{2}\right),\text{1}\text{e}-\text{5}\right)\text{ for }j=1,...,N_{i}$ and $s_{j}=min\left(max\left(N\left(s_{i},\text{0},\text{0}\text{5}\right),\text{0}\right),\text{1}\right)\text{ for }j=1,...,N_{i}$. Corresponding $r_{j}$ and $\varepsilon_{j}$ traits are then calculated using the trade-offs defined in [2] and [3].

All in all, the densities of plasmid-free and plasmid-i-carrying bacteria at time $t+\Delta_{t}$ are updated as:


$$\begin{array}{c} B_{\text{0}}\left(t+\Delta_{t}\right)=B_{\text{0}}(t)+G_{\text{0}}-L_{\text{0}}-\sum_{i=\text{1}}^{n}A_{i}+\sum_{i=\text{1}}^{n}\overset{―}{R_{i}} \end{array}$$

$$\begin{array}{c} B_{i}\left(t+\Delta_{t}\right)=B_{i}(t)+R_{i}-L_{i}+A_{i}-N_{i}\text{ for }i=\text{1},...,n \end{array}$$

$$\begin{array}{c} B_{j}\left(t+\Delta_{t}\right)=\text{1 for }j=\text{1},...,n^{\prime } \end{array}$$

### Simulating plasmid sampling in stochastic model

To compare simulated plasmid metacommunity densities (from the neutral and unified stochastic models, with millions of plasmids) to observed distributions (4,411 plasmids clustered in PTUs), we randomly down-sampled the simulated communities. For each community, we first determined how many plasmids to sample proportionally to the community’s plasmid-carrying bacteria. Within each community, the sampled plasmids were then distributed among species according to their relative frequencies. Finally, plasmid densities at the metacommunity scale were obtained by summing the sampled densities across all communities. This approach allows direct comparison between simulated and empirical plasmid abundance distributions while accounting for differences in total sample size.

### Measuring plasmid diversity

We characterized plasmid communities using three complementary indices. **Richness** was simply the number of plasmid species (S) in a sample. **Diversity** was quantified with the Gini–Simpson index, $S_{index}=\text{1}-\sum_{i=\text{1}}^{S}p_{i}^{\text{2}}$, where $p_{i}$ is plasmid *i* frequancy and which measures the probability that two randomly chosen plasmids belong to different species. This index approaches 0 when one species dominates and 1 when many species are rare. Finally, to capture trait-based diversity, we used **Rao’s functional index**, defined as $R_{index}=\sum_{i=\text{1}}^{S}\sum_{j=\text{1}}^{S}\Delta_{i,j}p_{i}p_{j}$ where $\Delta_{i,j}$ is the functional dissimilarity (measured by Chebyshev distance) between plasmids i and j. This measure increases with both the number of species and their trait dissimilarities, and is maximized when distinct species are abundant.

### Inference of dispersal and innovation rates

For both neutral and unified models of plasmid dynamics, we inferred the dispersal and innovation rates by exploring a parameter space of 49 combinations, crossing 7 dispersal rates with 7 innovation rates. We simulated 25 trajectories for each combination. The selected rates were those that minimized the mean deviance between the simulated and observed RACS from *E. coli* (presented in Fig 1A). Following classical definition, the deviance was calculated as


$$\begin{array}{c} d=\text{2}\left[\sum y_{observed}\text{ln}\left(\frac{y_{observed}}{y_{simulated}}\right)-\sum \left(Y_{observed}-y_{simulated}\right)\right] \end{array}$$

where $y_{observed}$ and $y_{simulated}$ are the sorted vectors of nonzero observed and simulated plasmid abundances, respectively.

## Supporting information

S1 Fig Plasmids diversity in *Klebsiella* and *Salmonella* genus. The first row **(A–B)** illustrates rank-abundance curve (RAC) of plasmid species (Plasmid Taxonomic Unit) for (A) the *Klebsiella* genus and (B) the *Salmonella* genus (using complete genomes from NCBI). Mirroring the patterns observed in *Escherichia coli* (Fig 1), these two plasmid communities exhibit similar abundance patterns: a few dominant species coexist with many moderately represented ones and a significant number of extremely rare species. These distributions were fitted using the Power-bend model (predicted abundances and 95% IC, see Materials and methods) which consistently outperformed five other classical species-abundance models (S1 Table). The second row **(C–D)** illustrates the distribution of plasmid length in (C) *the Klebsiella* genus and **(**D) the *Salmonella* genus. We tested the multimodality with a Gaussian Mixture Model (ranging from 1 to 7 components) which identified two mixed Gaussian as the best fit for both size distributions (see Materials and methods). The data and code required to generate this Figure can be found in https://doi.org/10.5281/zenodo.21220021.
(TIFF)

S2 FigPlasmid performance under fluctuating environments and plasmid competition under constant environment.**(A) Plasmids performance under fluctuating environments and without competition.** (Left column) $p_{res}$ mean density (${\text{log}}_{\text{1}\text{0}}$) calculated over the last period of antibiotic stress in the absence of competition with $p_{sens}$. (Right column) $p_{sens}$ mean density (${\text{log}}_{\text{1}\text{0}}$) calculated over the last period of antibiotic stress in the absence of competition with $p_{res}$. **(B)** Calculating the slope of plasmid density to estimate its persistence. To estimate if $p_{res}$ and $p_{sens}$ can coexist under fluctuating environments, we calculate the slope of the line connecting the min densities of each plasmid over each stress period (on a ${\text{log}}_{\text{1}\text{0}}$scale). In this example, the slope associated with $p_{sens}$ is negative, meaning that this plasmid is going extinct and therefore does not coexist with $p_{res}$ in these conditions. **(C–D)** Competition between plasmid species in constant environments. In both scenarios, 200 plasmid species are defined with varying traits. (C) Under continuous antibiotic stress, plasmids have an intermediate HTR ($\alpha_{i}=\text{1e}-\text{6}\forall i\in \text{1},...,\text{200}$) with intermediate vertical transmission probability ($\varepsilon_{i}=\text{0},\text{9}\text{5}\forall i\in \left\{\text{1},...,\text{2}\text{0}\text{0}\right\}$) and varying RLs from 0 to 1 ($s_{i}=\frac{i}{\text{2}\text{0}\text{0}}\forall i\in \left\{\text{1},...,\text{2}\text{0}\text{0}\right\}$); fitness cost $r_{i}$ is defined by equation (2). (D) In the absence of antibiotic stress, plasmids vary in their HTR ($\alpha_{i}=\text{1}\text{e}-\left(\text{7}+\text{2}\frac{\left(i-\text{1}\right)}{\text{1}\text{9}\text{9}}\right)\forall i\in \left\{\text{1},...,\text{2}\text{0}\text{0}\right\}$) and are all antibiotic-sensitive ($s_{i}=\text{0}\forall i\in \left\{\text{1},...,\text{2}\text{0}\text{0}\right\}$) with a low fitness cost ($r_{i}=\text{0},\text{9}\text{9}\forall i\in \left\{\text{1},...,\text{2}\text{0}\text{0}\right\}$); vertical transmission probability $\varepsilon_{i}$ is defined by equation (3). **Upper row (Single-species dynamics):** Equilibrium density of each plasmid species grown in isolation. These results demonstrate that, in the absence of competition, all defined plasmid species are capable of persisting under the considered conditions. **Center row (Community assemblage at different time points):** Temporal dynamics of the plasmid community when all species compete simultaneously. **Lower row (Invasion fitness):** Invasion fitness of each plasmid species when competing against the most fit species of the set. **In both scenarios (C and D),** these results illustrate that under constant environmental conditions, the plasmid species with the highest fitness eventually outcompetes all others. However, species with traits closely resembling those of the most competitive plasmid also exhibit relatively high fitness, leading to significantly slower competitive exclusion over time. Notably, competitive exclusion is faster under antibiotic stress (C) than in its absence (D). The data and code required to generate this Figure can be found in https://doi.org/10.5281/zenodo.21220021.(TIFF)

S3 Fig Neutral model sensitive analysis using diversity measures. **(A)** Inference of dispersal and innovation rates. We defined 49 parameter combinations by crossing 7 dispersal rates (ranging from 0 to 0.1) and 7 innovation rates (ranging from 0 to 1e−4). From these, we inferred the value of both dispersal and innovation rates used in Fig 3 (main text). These rates correspond to the combination that minimizes the deviance between simulated and observed Rank-Abundance Curves. Detailed deviance calculations are provided in the Materials and Methods section. **(B)** Plasmid richness (number of plasmid species). (Left column) Global plasmid metacommunity richness. We represent the mean richness over the 25 simulations. (Center column) Mean local plasmid community richness. For each of the 25 simulations, we first calculate each plasmid community richness, then we calculate the mean of these community richness. Finally we represent the mean of these means. (Right column) Plasmid community richness differences. For each of the 25 simulations, we calculate the community richness differences as the quotient of the global richness by the mean local richness. Finally, we represent the mean richness differences over 25 simulations. **(C)** Plasmid diversity (Gini-Simpson index, showing heterogeneity). (Left column) Global plasmid metacommunity diversity. We represent the mean heterogeneity over the 25 simulations. (Center column) Mean local plasmid community diversity. For each of the 25 simulations, we first calculate each plasmid community heterogeneity, then we calculate the mean of these community heterogeneity. Finally we represent the mean of these means. (Right column) Plasmid community diversity differences. For each of the 25 simulations, we calculate the community diversity differences as the quotient of the global diversity by the mean local diversity. Finally, we represent the mean diversity differences over 25 simulations. Panels B and C show how global and local richness and diversity increase with innovation and dispersal rates. The data and code required to generate this Figure can be found in −https://doi.org/10.5281/zenodo.21220021.
(TIFF)

S4 FigNeutral model with low horizontal transfer rate and high vertical transfer probability.Observed plasmid diversity (black dots) is compared with simulations. The median (blue curve) and interquartile (light blue area) densities for each rank are represented from 25 simulations. The mean deviance between observed and simulated data is computed (all others parameters are as defined in S3 Table and are the same as in Fig 3 in the main text). **(A)** When $\alpha_{i}=\text{1}.\text{0}\text{0}\text{0}\text{1}\text{e}-\text{7},\forall i\in \text{1},...,\text{2}\text{0}\text{0}$ and $\varepsilon_{i}=\text{0}.\text{9}\text{9}\text{9}\text{9}\text{9}\text{9},\forall i\in \text{1},...,\text{2}\text{0}\text{0}$**. (B)** When $\alpha_{i}=\text{1}.\text{0}\text{1}\text{e}-\text{7},\forall i\in \text{1},...,\text{2}\text{0}\text{0}$ and $\varepsilon_{i}=\text{0}.\text{9}\text{9}\text{9},\forall i\in \text{1},...,\text{2}\text{0}\text{0}$. The data and code required to generate this Figure can be found in https://doi.org/10.5281/zenodo.21220021.(TIFF)

S5 Fig Unified model temporal richness and diversity and sensitive analysis. **(A)** Inference of dispersal and innovation rates. We defined 49 parameter combinations by crossing 7 dispersal rates (ranging from 0 to 0.01) and 7 innovation rates (ranging from 0 to 10 − 4). From these, we inferred the optimal dispersal and innovation rates used in Fig 4 (main text). These rates correspond to the combination that minimizes the deviance between simulated and observed Rank-Abundance Curves (RACs). Detailed deviance calculations are provided in the Materials and Methods section. **(B)** Temporal evolution of plasmid richness and diversity. Dynamics are shown at the metacommunity scale (in black) and within the three distinct environments: high stress (in green), low stress (in blue) and intermediate stress (in pink). First row: Temporal evolution of plasmid richness (number of species). Second row: Temporal evolution of plasmid diversity (probability that two randomly sampled plasmids belong to different species). Third row: Temporal evolution of plasmid functional diversity (which captures the variance in plasmid traits: HTR and RL). Fourth row: Stress exposure pattern for each environment. After 1,000 generations, the community structure stabilizes across all scales. Species richness is highest in environments with rare or intermediate stress pulses. While functional diversity remains low within each local environment, it is markedly high at the metacommunity scale, reflecting the emergence of three distinct clusters specialized for each stress regime (see main text). Species diversity remains high in all environments due to the delayed competitive exclusion of highly similar species (see S3 Fig). **(C, D, E)** Sensitivity analysis of plasmid richness (C), diversity (D), and functional diversity (E). Indices are shown across three scales: global metacommunity (left column), local communities (center column), and inter-community differentiation (right column). **(C) Richness:** Both global and local richness increase with dispersal and innovation rates, leading to a decrease in richness differences between communities. (D and E) Diversity and Functional Diversity: Global diversity and functional diversity increase with the innovation rate. However, contrary to the neutral model, global diversity decreases as dispersal increases. High dispersal erodes the structuring effects of spatial heterogeneity, which favors a subset of dominant species, thereby reducing both global diversity and functional diversity. Because multiple species may possess identical or highly similar traits, dispersal—consistent with neutral model—can facilitate local coexistence. This increases local diversity without significantly affecting local functional diversity. The data and code required to generate this Figure can be found in https://doi.org/10.5281/zenodo.21220021.
(TIFF)

S6 FigUnified model with high vertical transfer probability ($\varepsilon_{min}=0.999,\varepsilon_{max}=0.999999$) and low horizontal transfer rate ($\alpha_{min}=1𝐞-15,\alpha_{max}=1𝐞-5$).**A.** Distribution of plasmid densities in the space of plasmid traits. Each dot represents a plasmid species in trait space (HTR on the x-axis, RL on the y-axis). Dot size reflects abundance; color indicates the community identity. (T = 0) Initial species traits are randomly distributed with equal abundances. (T = 1,000) After 1,000 generations, plasmids begin diverging toward peripheral strategies, high/low resistance, high/low transfer, or both. (T = 5,000) After 5,000 generations, three distinct clusters have emerged: (i) high RL with low HTR, (ii) high HTR with low RL, and (iii) high HTR with high RL. This reflects an eco-evolutionary sorting driven by local stress regimes and connectivity across communities. **B.** Observed plasmid diversity (black dots) is compared with simulations. The median (blue curve) and interquartile (light blue area) densities for each rank are represented from 25 simulations. The mean deviance between observed and simulated data is computed. The data and code required to generate this Figure can be found in https://doi.org/10.5281/zenodo.21220021.(TIFF)

S1 Table Table of RAC model fitting data (using *fitsad* function from R package *sads*). Across all datasets, the Pueyo’s power-bend model was selected as the best-supported model based on AIC, or showed equivalent support to the Fisher or Poisson models (ΔAIC ≤ 2).
(DOC)

S2 Table Variables and parameters of deterministic model [1]. Table listing the definition, dimension, value and justification of all variables and parameters in the deterministic model (Figs 2 and S2).
(DOC)

S3 Table Variables and parameters of the neutral model. Table listing the definition, dimension, justification and value of all variables and parameters in the neutral model (Figs 3, S3 and S4).
(DOC)

S4 Table Variables and parameters of the unified model. Table listing the definitions and values of all variables and parameters in the deterministic model (Figs 4, S5 and S6).
(DOC)

S1 Movie Available in the Zenodo archive (https://zenodo.org/records/21220021). The movie shows plasmid communities’ eco-evolutionary dynamics in time. **(Left panel)** Assembly of plasmid species. Each dot represents a plasmid species in trait space (HTR on the x-axis, RL on the y-axis). Dot size reflects abundance; color indicates the community identity. **(Right panel)** Plasmid community stress patterns. (Upper row) Communities 1–4 experience long frequent stress. (Intermediate row) Communities 5–8 experience short rare stress. (Lower row) Communities 9–12 experience intermediate stress. The data and code required to generate this Figure can be found in https://doi.org/10.5281/zenodo.21220021.
(AVI)

## Abbreviations

AIC: Akaike Information Criterion
BCI: Barro Colorado Island
BIC: Bayesian Information Criterion
CI: confidence intervals
HGT: horizontal gene transfer
HTR: horizontal transfer rate
MGEs: mobile genetic elements
RAC: rank-abundance curve
RACs: Rank-Abundance Curves
RL: resistance level.
