# Supplementary material for: Ecological theory sheds light on plasmid diversity and dynamics
Source: PLoS Biol. 2026 Aug 28;24(8):e3003918. doi: 10.1371/journal.pbio.3003918 (PMC13524261; doi:10.1371/journal.pbio.3003918)
Supplement: Supplementary file 1 [file pbio.3003918.s014.pdf]

Response to Reviewers

Plasmid diversity and dynamics in the light of ecological theories

Authors: Rémi Tuffet[1, 2, 3]*, Emma Acacia[1], Charles Coluzzi[1], Xavier Charpentier[2], Thomas Koffel[1]†, Samuel Venner[1]*†

Affiliations:

[1] Laboratoire de Biométrie et de Biologie Évolutive, Université Claude Bernard Lyon 1; Villeurbanne, 69100, France

[2] Centre International de Recherche en Infectiologie, Université Claude Bernard Lyon 1; Lyon 69007, France

[3] Current Institution: Institut d'écologie et des sciences de l'environnement de Paris, Sorbonne Université Campus Pierre et Marie Curie; Paris, France

† These authors contributed equally.

*Corresponding authors. Email: remi.tuffet@sorbonne-universite.fr or samuel.venner@univ-lyon1.fr

Introduction to the Response to Reviewers

In the following, reviewers' comments are indicated in **black** (exactly as written), and our responses are provided in **blue**.

We would like to thank the three reviewers for their insightful and constructive comments. To address the main points raised by several reviewers, we have structured this response with a global common response outlining the major revisions made to the manuscript. Our responses are indexed (R0.Y for the global response and RX.Y for point-by-point responses) for easy cross-referencing.

Global common response

R0.1: Reframing the introduction and theoretical context. Following the reviewers' suggestions that our work was not sufficiently positioned within the existing literature, we have significantly revised the Introduction. We now explicitly refer to the "plasmid paradox" and incorporate key contributions regarding the maintenance of plasmids and the diversity of their traits (l. 57-71). We emphasize that plasmids interact not only with their bacterial hosts but also with one another, allowing us to treat plasmids as a community of competing entities. While this conceptual shift has been suggested for transposable elements and other MGEs, we argue that a robust ecological framework is now essential to quantify the relative importance of the processes driving plasmid eco-evolutionary dynamics.

R0.2: Comparative analysis of diversity patterns: plasmids vs. birds and trees. To demonstrate that plasmid diversity patterns are comparable to those described in ecology, we have replaced our initial visual descriptions with rigorous statistical analyses (Fig. 1 and S1 and Table S1).

- Rank-Abundance Curves (RAC): We have corrected our terminology (replacing "Species Abundance Distribution" with "Rank-Abundance Curve"). We compared the performance of

six classical ecological models to fit the RACs. Using the Akaike Information Criterion (AIC), we show that Pueyo's power-bend model is the best fit for plasmids, birds, and trees alike, suggesting that similar underlying processes govern these disparate systems.

- Size Distribution: To rigorously identify the multimodality of traits, we employed Gaussian Mixture Models (GMM) and included the best-fit distributions in the figures.

The Materials and Methods section has been expanded to detail these new statistical procedures (l.543-555)

R0.3: Generalization to others genera (*Klebsiella* and *Salmonella*). To strengthen our proposition, we now show that the diversity patterns observed in *E. coli* are consistent across the *Klebsiella* and *Salmonella* genera—the only other genera with sufficient plasmid data in public databases (3,277 and 1,168 clustered plasmids, respectively) to allow for robust analysis. These results are now presented in Figure S1, while the main text remains focused on the *E. coli* dataset.

R0.4: Inference of dispersal and innovation rates. We have clarified how dispersal and innovation rates were estimated for both the neutral and unified models. We explored a parameter space of 49 combinations (7 dispersal rates × 7 innovation rates) and selected the combination that best fits the observed *E. coli* RACs for each model. This methodology is detailed in the Materials and Methods section (l. 651-659) and the results are presented in the Supporting Information (Figs. S3A, S4A). Consequently, we have expanded the Results section (l. 278-281 and l. 361-368) and figure legends (fig S3 & S4) to provide a more in-depth description of how these rates influence plasmid richness and diversity (sensitivity analysis).

R0.5: Strengthening the Discussion. We have extensively revised the Discussion to better articulate how community ecology informs our understanding of plasmid diversity. Specifically, we added a paragraph discussing how neutral theory can be extended by considering horizontal transfer between bacterial species. We also discuss niche-based extensions, such as resource partitioning and apparent competition.

R0.6: Technical Revisions and Parameter Justification. All parameter tables (Tabs. S2, S3, and S4) have been revised to include parameter dimensions and detailed justifications for their selected values.

As a result of these substantial improvements, the length of the manuscript has increased significantly, providing a more comprehensive and robust study.

Reviewer 1 comments

In this manuscript, the authors argue how plasmids (or mobile elements in general) could be studied through the lens of classic ecological theories, while also introducing an extended model to include multiple relevant processes acting on (the diversity of) plasmids. The manuscript is well written, and I enjoyed their perspective very much. There are a few points where I would argue the strength could be improved, or perhaps where I simply got confused.

First some broader, general remarks. I very much enjoyed the intro (very lovely written!) and the analysis of figure 1. However, in discussing figure 2 and 3 I feel like the authors are somewhat missing the opportunity to emphasise that these classical models (determinism vs. neutral) are a bit of a false dichotemy to begin with. Neutrality and selection are heavily variable under most reasonable conditions in nature, and indeed figure 4 reeled me back in by putting aside this black-and-white thinking. I found myself a little frustrated during the part of figure 2 and 3, which in the end wasn't needed as figure 4 brought all the nuance back. Perhaps some foreshadowing of what will be built towards could have avoided this frustration, which I think other readers may also experience.

R1.1: We agree. We have revised the introduction to the modeling section (titled "Applying biodiversity theories to plasmids" l.170-182) accordingly to emphasize that our model allows us to assess the relative importance of various ecological processes, and that our presentation is structured in a step-by-step manner to facilitate an understanding of each process and an examination of their significance (before considering them all together).

One other general remark is that people have recently studied similar patterns in other MGEs (phages, IS elements, e.g. Flora Gaudilliere's work on transposons in prokaryotes https://www.biorxiv.org/content/10.1101/2025.07.22.666184v1.full.pdf+html), so I'd at least acknowledge that this way of thinking is already happening. That's not to say you're not doing something unique, but I would think it's better to frame this as a new way that people are thinking about this topic, rather than as a pioneering view.

R1.2: In response to this comment and a similar one from Reviewer 3, we have completely rewritten the introduction (l.55-102) to better contextualize our proposal within the existing body of work (see also A0.1). We do this into two parts:

1) We emphasize that the vast majority of studies addressing the evolution of plasmid strategies focus on the relationship between plasmids and their bacterial hosts and attempt to resolve the plasmid paradox
2) We emphasize that studies seeking to apply the theoretical framework of community ecology to understand the diversity and assembly of MGEs are rarer. We make clear reference to some of these studies (including (Gaudillière *et al.*, 2025)). We also note that our work proposes a rigorous framework to help lay the groundwork for the development of a unified theory of plasmid diversity and, more generally, of MGEs.

Just a positive remark: that supplementary video is absolutely lovely, and it's illustrating a pretty cool result :)

Happy to look at your revised manuscript if the editor wishes, it's a really fun and light read :)

We are delighted to hear that the supplementary video was useful and sincerely thank the reviewer for their kind remarks and positive assessment of the manuscript.

<u>Some smaller points:</u>

- Abstract: comma after "yet" missing. (double check me on this, I feel like it should be followed by a comma but I could be wrong)

R1.3: Corrected l.36.

- Personal preference, but in figure 1 caption / it's discussion in the text, I would personally not use "striking" here, as these patterns are indeed the default behaviour from neutral models (as you explain later), so any DEVIATION from this pattern would be striking. I would instead simply emphasise the similarity without any adjectives.

R1.4: We agree: the words "striking" and "strikingly" were removed from the main text and Figure 1 caption.

- At the bottom of page 4 "i.e. spread in the bacterial population, when alone exploiting..." this sentence structure is confusing. I would move the "alone" part forward in the sentence, e.g. "... each plasmid can grow when alone, i.e. spread through the bacterial population, in the absence of competition by the other." (or sth like that)

R1.5: We agree, the sentence was corrected (l.191-193) as follows:

"Under the two antibiotic stress conditions illustrated, each plasmid species can grow when alone, i.e. spread through the bacterial population, in the absence of competition from the other (dotted lines in Fig. 2B-C). "

- Figure 3 A: why did you go to red-green when figure 2 had blue-red plasmids? I am red-green colour blind, so I appreciated the colours in 2 more.

R1.6: We agree that the choice of red in the diagram in Figure 3 is problematic because this color may suggest that the properties of the red plasmids are the same as those in Figure 2. On the contrary, in Figure 3, which illustrates the neutral theory, all plasmid variants are equivalent in their traits. In this figure, we have chosen to replace the different colors of the plasmids with different shades of gray to eliminate any ambiguity.

- Figure 3 B: I would not use viridis-colours (a continuous scale) for displaying what I think are categorical differences of plasmid species.

R1.7: In this figure, the choice of a viridis palette color is constrained by the high number of different plasmid species ($N = 200$) to represent. Although we agree that using a continuous scale for displaying categorial differences is not ideal, in our context we believe this is the easiest and clearest way to do. However, if other options that we had not thought of seem better adapted, we are ready to make the changes.

- Figure 3 D: is the effect of dispersal monotonic? Is higher dispersal always leading to higher diversity, or can it also go down again? (I think not, as your model assumes only bacteria disperse while environments stay heterogeneous? perhaps a supplementary figure on how dispersal shapes diversity would be nice)

R1.8: Thanks for this relevant question which echoes concerns raised by Reviewer 2 regarding the insufficient integration of our supplementary figures (now Figs. S3 and S4) in the initial version.

In the Neutral Model: Indeed, the effect of dispersal is monotonic. As shown in Figure S3B-C, where we systematically explore the parameter space for both innovation and dispersal rates, we observe that for any fixed innovation rate, both richness and diversity (Gini-Simpson index) increase with dispersal rate. This occurs because higher dispersal limits local extinction events by promoting the rescue effect across the metacommunity. We have updated the Results section (l. 278–281) and the legend of Figure S3 to clarify this point.

In the Unified Model: The dynamics are more complex and non-monotonic. In this context, we consider three distinct environmental regimes. While intermediate dispersal rates promote diversity by allowing plasmids to reach suitable niches, very high dispersal rates tend to homogenize the metacommunity. This 'swamping effect' overrides the structuring influence of spatial heterogeneity, eventually leading to the exclusion of specialists and a decrease in overall diversity. These patterns are now detailed in the Results section (l. 361–368) and the legend of Figure S4.

We believe these additions provide a more nuanced understanding of how dispersal interacts with environmental selection to shape plasmid assemblages.

I may have missed it but are the parameters choices mentioned anywhere else but in the supplementary file? Is the fit of the data shown in figure 3/4 using inferred parameters? Wasn't completely clear to me.

R1.9: Indeed, parameter values are not discussed in the main text. These values are listed in Tab. S2, S3, and S4. Although most of these values come from the literature, no justifications were given. To address this, we added a column in these tables to justify our choices.

In contrast, innovation and dispersal rates were inferred to best fit the observed data. We measured the deviance between simulated and observed data and selected the couple of parameters for which this value was minimal. We added in Fig. S3 and S4 the panel A showing a heatmap of deviance as a function of dispersal and innovation rate in the case of neutral model and unified model, respectively. The method is also described in the corresponding captions.

- Methods: I am not entirely sure why the vertical transmission term (Ei) looks so complicated with the log(alpha) stuff. It's probably obvious in hind sight but I can't see it now, perhaps make sure you explain why that choice was made / why it's biologically sound.

R1.10: We agree with Reviewer 1 that our parametrization of the vertical transfer term ε_i is far from intuitive. As a reminder, we computed ε_i as $\varepsilon_i = \varepsilon_{max} + (\varepsilon_{min} - \varepsilon_{max})\frac{\alpha_i'}{\alpha_{max}}$ where α_i' is a transformation on a log_{10} scale of the horizontal transfer rate α_i defined by $\alpha_i' = \alpha_{max}\frac{log_{10}(\alpha_i) - log_{10}(\alpha_{min})}{log_{10}(\alpha_{max}) - log_{10}(\alpha_{min})}$, with α_{min} and α_{max} the minimal and maximal horizontal transfer rates, respectively. Using a log scale allows us to consider plasmids with horizontal transfer rates ranging on several orders of magnitude, in accordance with the literature (Sheppard, Beddis and Barraclough, 2020; Quon *et al.*, 2025). We logically then also express the cost of this trait on the vertical transfer parameter on this same log_{10} scale. We now give more details on this in Materials and Methods (l. 588-596). Note that no study has yet been able to empirically characterize this trade-off between horizontal and vertical transfer. We thus also emphasize that one perspective emerging from our work is the need to better characterize this trade-off.

Reviewer 2 comments

In this manuscript, Tuffet and colleagues consider plasmid distributions using established ecological frameworks developed to understand animal and plants. They consider how plasmid 'species' distributions in a database of E. coli genomes mirrors those of animals and plants, and develop a general mathematical model of ecological processes. The model yields results consistent with these patterns, with the main conclusion highlights the role that neutral processes could play in determining plasmid distributions. Overall this was a nicely written and presented paper. The key result — that neutral models do a reasonable job of capturing patterns of plasmid distribution — is

valuable and somewhat provocative, since many people assume that plasmids are driven principally by selection for specific accessory traits. More than this, the application of these ecological frameworks to mobile genetic elements dynamics is pioneering and makes a valuable contribution to the literature, and an exemplar for future studies in this area. Even if the model is relatively simple, and the key results somewhat obvious, I think this work has the potential to make an important impact. However, I have some major concerns with the way that the data are interpreted, principally owing to the biases in the plasmid database.

We thank Reviewer 2 for their overall positive review.

<u>Main issues/comments:</u>

- The work focuses on E. coli and its cognate plasmids, and generates its database using E. coli genomes from NCBI RefSeq. Focusing on a single species is sensible, but I'm concerned that the sampling of strains that ultimately yield the genomes on RefSeq will have been highly biased towards human and animal pathogens, resulting in repeating sampling of epidemic clones. For example, ST131 is a dominant pandemic clone that frequently harbours plasmids. Consequently, I can envisage how this kind of biased sampling might produce a species abundance distribution of the kind described in Figure 1A simply as a consequence of a particular pathogenic clone being more predisposed to a particular plasmid or PTU, and 'rarer' plasmids being found fewer times just because their hosts are less of a concern (and therefore less likely to be sequenced) than pathogens. There have been some attempts to control for this kind of correlation, which the authors might consider, e.g. as described by

Dewar et al. 2021 10.1038/s41559-021-01573-2. Another approach (perhaps less robust) would be to downsample their data to a single clone per BioProject, to ensure that their results are not dominated by repeated sampling of a clonal outbreak. But the authors need to make some effort to demonstrate that biased sampling in the E. coli dataset is not the driver of their observations.

R2.1. We thank Reviewer 2 for their very relevant comment and for pointing us toward the work of (Dewar *et al.*, 2021). We fully agree that sampling bias and the over-representation of pandemic clones in public databases could potentially skew plasmid abundance distributions. To address this concern, we have refined our methodology and expanded our analyses:

- **Stringent Strain De-replication:** Following the logic proposed by Dewar *et al.* (2021) to control for clonal expansions, we implemented a sequence-based de-replication step to ensure genomic independence. While Dewar *et al.* (2021) effectively used strain nomenclature to identify lineages, we chose to apply a more robust approach based on a genomic distance. Genomes with a MASH distance below 0.0001 were clustered, and only a single representative genome from each group was retained. This sequence-based threshold ensures that our dataset is not dominated by pseudo-replicates of epidemic clones, even in cases where strain metadata might be incomplete. We have clarified this procedure in the revised Materials and Methods (l. 501-506) to better highlight how we mitigated clonal over-representation.
- **Generalization across Genera:** To further ensure that our observations are not an artifact of *E. coli* population structure, we extended our analysis to two other major enteric genera: *Klebsiella* and *Salmonella* (see Global Response **A0.3**). As shown in the new **Figure S1**, we observed qualitatively identical patterns in both the Rank-Abundance Curves and size distributions across these three genera. The consistency of these results across different

bacterial groups suggests that the observed plasmid diversity is driven by fundamental eco-evolutionary processes rather than sampling biases specific to *E. coli*.

- How are the models parameterised? The authors include tables of values (Tables S1-3) but these are not well described or justified. I'm not sure whether the regimes presented are ecologically relevant, nor am I sure of the sensitivity of the results to the selected parameters.

R2.3. We acknowledge that the justification for our parameter choices and the assessment of model sensitivity were not sufficiently highlighted in the initial version of the manuscript. We have revised our work as follows:

- **Parameter Documentation:** Tables S2–S4 have been revised to include the dimensions of all parameters and more explicit justifications for their values. For the dispersal and innovation rates used in the neutral and unified models, we have clarified that these were not fixed *a priori* but were **inferred** to best fit the observed *E. coli* Rank-Abundance Curves (see Global Response **R0.4**).
- **Sensitivity and Robustness:** We have added a more detailed description of the sensitivity of our results to variations in dispersal and innovation rates. These analyses are now extensively discussed in the Results section (l.278-281 and l. 361-368)
- as well as in the legends of **Figures S3 and S4**. (see also R1.8 & R1.9)
- **Conceptual Framework for Stress Regimes:** We now clarify that the stress regimes are theoretical rather than empirical. They are designed as a heuristic tool to represent spatiotemporal heterogeneity and to illustrate how niche partitioning can stabilize plasmid coexistence. This framework allows us to show, at a conceptual level, how niche and neutral theories can be unified. We have also expanded the Discussion section to address other potentially stabilizing mechanisms and factors that may influence coexistence in natural systems.

There appears to be some sensitivity analysis in Figs. S2 and S3 but these are not well described. More detail needs to be provided here, and an additional column in the table of parameter values justifying the selected range for each term with reference to the literature.

R2.4. We agree with Reviewer 2's comment. These sensitivity analysis figures (now Fig. S3 and S4) should now be better described. First, we completed their captions to explain them better. Second, we now discuss these figures in the corresponding Result section to highlight their main messages (See also R2.3).

- The first conclusion in the results is that plasmid abundance patterns 'mirror' those of animals and plants, a conclusion that is based on visual inspection of rank (log) abundance curves. It is a bit unsatisfying to rest the conclusion on this somewhat subjective 'eyeball' analysis. It would be valuable to formally test how similar these curves are. This might be done with a two-one-sided test variant of the Kolmogorov-Smirnov test, some kind of bootstrapping/permutation test, or there may be a more elegant approach that I am not familiar with.

R2.5. We agree that a visual "eyeball" analysis was insufficient for such a central claim. In this revised version, we have moved beyond subjective inspection by implementing a rigorous statistical framework to test the similarity between plasmid, bird, and tree forest community patterns:

- Model Selection for RACs: Rather than simple visual comparison, we performed formal model fitting on the Rank-Abundance Curves (RACs) for plasmids, birds, and trees using six classical ecological distributions. As detailed in Global Response A0.2, we used the Akaike Information Criterion (AIC) to identify the best-fit model for each dataset. Crucially, we found

that the same mathematical model (Pueyo's power-bend) provides the best fit across all three disparate systems, supporting the idea that they are governed by a similar class of eco-evolutionary processes.

- Trait Multimodality: To compare trait distributions (plasmid size vs. body mass/height), we employed Gaussian Mixture Models (GMM) combined with the Bayesian Information Criterion (BIC) to objectively identify the number of modalities. This allowed us to formally demonstrate that plasmid size distributions exhibit a multimodality analogous to that observed in ecological assemblages.

We believe these quantitative analyses provide the "formal test" requested and offer a much more robust proof of the parallels between plasmid communities and ecological systems.

<u>Minor comments:</u>

- Figure S1 panels C and D are difficult to interpret. More information should be provided in the legend to explain what these are showing.

R2.6. We agree. Following Reviewer 2's suggestion, we now provide more information in the legend to explain the figures and what they show.

- On the subject of selection vs. neutral processes in plasmid abundance patterns, the authors might like to touch on the recent paper by Adrian Cazares and colleagues (2025) that describes plasmids from pre-antibiotic era strains, which suggests a greater diversity of plasmids from the pre-antibiotic era. I wonder how SADs would be affected by strong selection on a selected subset of plasmids?
R2.7. We thank Reviewer 2 for the excellent suggestion to include the recent work by Cazares *et al.* (2025) which is indeed highly relevant to our findings as it provides a unique longitudinal view of plasmid evolution. Based on their findings, we have refined our discussion to move beyond simple richness and focus on how selective pressures reshuffle plasmid assemblages. We have integrated this into the manuscript through the following modifications:

- **Analysis of RACs by environmental regime:** In the results of our unified model, we now detail the Rank-Abundance Curves (RACs) across different environmental fluctuation regimes (Fig. 4B). We show that while intense selection favors the emergence of specific specialized plasmids, it also accelerates the competitive exclusion, shifting the community structure (richness diminution).
- **Mechanistic explanation in results:** We clarified that intense antibiotic pressure accelerates competitive exclusion relative to stress-free conditions (as illustrated in **Fig. S2 C-D**), which directly reduces local species diversity. This process provides a mechanistic basis for understanding why certain 'ancestral' plasmid lineages might disappear when faced with strong deterministic selection.
- **Discussion of pre- vs. post-antibiotic era:** In the Discussion, we used these results to provide a mechanistic perspective on the dynamic processes under antibiotic pressure. We discuss how the industrialization of antibiotics has favored the emergence of large, complex multidrug-resistance plasmids while simultaneously altering the composition of the 'long tail' of rare plasmids. We also highlight that our model predicts different diversity patterns depending on the intensity of local selective pressures, which remains a key area for future empirical investigation. We added the following paragraph to the discussion (l.447-456):

  "Our analysis of plasmid diversity across heterogeneous environments reveals that intense antibiotic pressure does not merely favor the emergence of specific plasmids; it also

accelerates competitive exclusion, thereby reducing local species richness (Fig. 4B, S2). These findings may help clarify the observed shifts in plasmid diversity between the pre-antibiotic era and the modern clinical landscape (Cazares et al., 2025): while the widespread use of antibiotics has favored the emergence of conspicuously large and complex modern plasmids, often through fusion and multi-drug resistance acquisition, it likely altered the composition of the broader 'long tail' of rare plasmids. However, our results also indicate that these diversity patterns should vary significantly depending on local selective pressures (e.g., low vs. high antibiotic exposition), a prediction that remains to be further investigated in contemporary plasmid communities."

We believe these additions significantly strengthen the paper by bridging our theoretical predictions with recent empirical data on plasmid turnover and evolution.

- It would be interesting also to consider how plasmid abundance patterns are affected by the possibility of resistance gene capture, i.e. where a plasmid is lost and the resistance genes migrate to the chromosome. Perhaps this is similar to a very low conjugation rate? Might be worth a couple of words in the discussion, since 'chromosomal capture' is a key principle of the plasmid paradox.
R2.8. We thank Reviewer 2 for their comment. Integrating the concept of chromosomal capture indeed allows us to further bridge our findings with the "plasmid paradox" and the long-term evolution of resistance. Following Reviewer 2's suggestion, we have added a discussion of this mechanism as a logical outcome of intense selection:

- **Integration into the Discussion:** We now address chromosomal capture in the context of our third plasmid cluster (high vertical transmission/low HTR), which emerges under chronic stress. We argue that while some plasmids adapt by reducing their fitness cost (e.g., through compensatory mutations), others may reach an evolutionary dead-end where chromosomal integration becomes the most parsimonious solution.
- **Revised text (l. 441-446):** " Finally, chronic stress favors plasmids with high vertical transmission (low HTR) and high resistance. This latter group mirrors plasmids that have minimized their fitness cost, for example through compensatory mutations (Liu *et al.*, 2024). If, however, the plasmid remains significantly costlier under such persistent pressure, these conditions might ultimately favor the chromosomal capture of resistance genes, thereby eliminating the metabolic burden of plasmid maintenance while preserving the adaptive trait (Bergstrom, Lipsitch and Levin, 2000). "

We believe this addition provides a more comprehensive view of the potential outcomes of plasmid-host coevolution under strong deterministic selection.

Reviewer 3 comments

In the manuscript "Plasmid diversity and dynamics in the light of ecological theories", the authors argue that plasmids can be viewed as ecological entities competing for bacterial hosts. This is a compelling perspective that could have important implications for how we understand plasmid persistence, diversity, and their role in bacterial evolution, including the spread of antibiotic resistance. However, earlier theoretical studies have explored this perspective in various forms for decades, and contemporary work continues to refine it. For example:

10.1086/416582 (Eberhard 1990)

10.1007/BF02540223 (Simonsen 1991)

10.1038/nrmicro1643 (Prosser 2007)

10.1016/j.ecolmodel.2025.111418 (Alderliesten 2026)

10.48550/arXiv.2509.01695 (Nauta preprint)

R3.1. We thank Reviewer 3 for bringing these references to our attention. We completely revised our Introduction to better contextualize our work within the long-standing theoretical history of plasmid biology. We now explicitly cite the seminal works of (Eberhard, 1990) and (Simonsen, 1991), alongside the more recent perspectives of (Prosser *et al.*, 2007), (Alderliesten *et al.*, 2026) and (Nauta *et al.*, 2025). By integrating these references, we have clarified that while the "plasmid paradox" has indeed been a central theme for decades, our study's originality lays in shifting the focus from the persistence of a single plasmid-host pair toward the mechanisms maintaining the global diversity of plasmid assemblages.

The manuscript is explicitly framed as a bridge between plasmid biology and ecological theory. This interdisciplinary ambition is appealing, but it also raises the question of what genuinely new insight is provided to either community. From a plasmid biology perspective, the ecological framing is elegant but largely rearticulates mechanisms that have been extensively explored in prior theoretical and empirical work, including competition, trade-offs between horizontal and vertical transmission, environmental heterogeneity, stochastic drift, dispersal, and innovation. As such, the study does not clearly yield new plasmid-specific mechanisms or actionable predictions that would change how plasmid biologists think about plasmid persistence, diversity, or resistance evolution.

R3.2. While we appreciate this critique, we respectfully disagree with the idea that our study only "rearticulates" existing mechanisms. While individual mechanisms like competition or horizontal transfer are well-known, they have almost exclusively been studied to explain the maintenance of a specific plasmid (the "paradox"). Our study is the first to our knowledge to formally demonstrate that the global distribution of plasmid abundance (the Rank-Abundance Curve) can be quantitatively explained by a unified eco-evolutionary model. We provide a robust framework that explains the coexistence of dozens of plasmid species within pangenomes, showing that this diversity is not random but structured by the interplay between niche partitioning and neutral drift. This provides a new, actionable way for plasmid biologists to interpret pangenomic data—shifting from a list of traits to a dynamic community assembly perspective. We have expanded the Discussion to highlight these specific insights.

From an ecological perspective, the work primarily applies well-established concepts, including neutral and niche theory, emergent neutrality, species abundance distributions, and metacommunity dynamics, to plasmid systems. While plasmids provide an interesting and tractable biological example, the manuscript does not appear to extend, challenge, or refine ecological theory itself, but rather illustrates its applicability in a new domain. Consequently, it is unclear whether ecologists would view this study as offering conceptual advances, as opposed to a compelling case study.

R3.3. The reviewer correctly identifies that our primary goal is to introduce plasmid researchers to ecological frameworks. We agree that our study is not intended to "challenge" ecological theory itself, but rather to show its universal applicability using a unique biological system, mobile genetic entities. Yet, we believe that applying ecological theory to plasmids is more than just a "case study." It offers ecologists new, high-throughput testing grounds where generations pass in hours and metacommunities are composed of billions of hosts. We now highlight in the Discussion (l. 482-497) the specificities of MGEs (e.g., their nested nature within host genomes) that could, in future work,

lead to novel refinements of ecological theory. We chose to keep this focused to maintain a clear message for our primary audience: biologists interested in the evolution of microbial genomes.

In this sense, the manuscript reads primarily as a conceptual synthesis that successfully reframes existing ideas across disciplines, but does not yet clearly deliver new biological insight for plasmid research nor new theoretical insight for ecology. Clarifying what is genuinely learned on either side of this interface, beyond the demonstration that ecological frameworks can be applied to plasmids, would substantially strengthen the contribution. Absent such clarification, the work may be better suited for a more ecology-focused or conceptual journal, where its integrative perspective would be appreciated as synthesis rather than as a primary biological advance.

R3.4. We have significantly strengthened the Introduction and Discussion to move beyond conceptual synthesis. To our knowledge, no other study has combined: (i) a rigorous statistical comparison of plasmid diversity patterns with ecological systems (birds, trees), (ii) a multi-genus validation (*E. coli*, *Klebsiella*, *Salmonella*), and (iii) a unified model that predicts the impact of antibiotic stress on species richness. For example, by demonstrating that antibiotic pressure accelerates competitive exclusion and "prunes" the long tail of plasmid diversity, our work offers a mechanistic explanation for historical shifts in plasmid pangenomes (e.g., pre- vs. post-antibiotic era). We believe that this combination of empirical data analysis and unified modeling constitutes a primary biological advance that lays the groundwork for a new "Ecology of MGEs".

References

Alderliesten, J.B. *et al.* (2026) 'The effects of ecological interactions and distinct conjugation rates on the invasion of a conjugative plasmid in bacterial communities', *Ecological Modelling*, 512, p. 111418. Available at: https://doi.org/10.1016/j.ecolmodel.2025.111418.

Bergstrom, C.T., Lipsitch, M. and Levin, B.R. (2000) 'Natural Selection, Infectious Transfer and the Existence Conditions for Bacterial Plasmids', *Genetics*, 155(4), pp. 1505–1519. Available at: https://doi.org/10.1093/genetics/155.4.1505.

Cazares, A. *et al.* (2025) 'Pre- and postantibiotic epoch: The historical spread of antimicrobial resistance', *Science*, 390(6777), p. eadr1522. Available at: https://doi.org/10.1126/science.adr1522.

Dewar, A.E. *et al.* (2021) 'Plasmids do not consistently stabilize cooperation across bacteria but may promote broad pathogen host-range', *Nature Ecology & Evolution*, 5(12), pp. 1624–1636. Available at: https://doi.org/10.1038/s41559-021-01573-2.

Eberhard, W.G. (1990) 'Evolution in Bacterial Plasmids and Levels of Selection', *The Quarterly Review of Biology*, 65(1), pp. 3–22. Available at: https://doi.org/10.1086/416582.

Gaudillière, F. *et al.* (2025) 'Unwelcome guests: characterizing the ecological niche of insertion sequences within prokaryotic genomes'. bioRxiv.

Liu, Z. *et al.* (2024) 'Compensatory evolution of chromosomes and plasmids counteracts the plasmid fitness cost', *Ecology and Evolution* [Preprint]. Available at: https://doi.org/DOI:%2010.1002/ece3.70121.

Nauta, J. *et al.* (2025) 'Heterogeneity drives plasmid maintenance in large microbial communities'. arXiv. Available at: https://doi.org/10.48550/arXiv.2509.01695.

Prosser, J.I. *et al.* (2007) 'The role of ecological theory in microbial ecology', *Nature Reviews Microbiology*, 5(5), pp. 384–392. Available at: https://doi.org/10.1038/nrmicro1643.

Quon, H. *et al.* (2025) 'Quantifying conjugation rates in clinical and environmental matrices: a systematic review to inform risk assessment', *Frontiers in Microbiomes*, 3, p. 1490240. Available at: https://doi.org/10.3389/frmbi.2024.1490240.

Sheppard, R.J., Beddis, A.E. and Barraclough, T.G. (2020) 'The role of hosts, plasmids and environment in determining plasmid transfer rates: A meta-analysis', *Plasmid*, 108, p. 102489. Available at: https://doi.org/10.1016/j.plasmid.2020.102489.

Simonsen, L. (1991) 'The existence conditions for bacterial plasmids: Theory and reality', *Microbial Ecology*, 22(1), pp. 187–205. Available at: https://doi.org/10.1007/BF02540223.
