# Supplementary material for: Ecological theory sheds light on plasmid diversity and dynamics
Source: PLoS Biol. 2026 Aug 28;24(8):e3003918. doi: 10.1371/journal.pbio.3003918 (PMC13524261; doi:10.1371/journal.pbio.3003918)
Supplement: Supplementary file 2 [file pbio.3003918.s015.pdf]

Response to Reviewers

Ecological theory sheds light on plasmid diversity and dynamics

Authors: Rémi Tuffet[1, 2, 3]*, Emma Acacia[1], Charles Coluzzi[1], Xavier Charpentier[2], Thomas Koffel[1]†, Samuel Venner[1]*†

Affiliations:

[1] Laboratoire de Biométrie et de Biologie Évolutive, Université Claude Bernard Lyon 1; Villeurbanne, 69100, France

[2] Centre International de Recherche en Infectiologie, Université Claude Bernard Lyon 1; Lyon 69007, France

[3] Current Institution: Institut d'écologie et des sciences de l'environnement de Paris, Sorbonne Université Campus Pierre et Marie Curie; Paris, France

† These authors contributed equally.

*Corresponding authors. Email: remi.tuffet@sorbonne-universite.fr or samuel.venner@univ-lyon1.fr

Introduction to the Response to Reviewers

We thank the editorial team and the reviewers for their constructive feedback on this revised version of our manuscript. In the following text, the reviewers' comments are indicated in black, and our responses are provided in blue.

Following this new round of review, we have introduced several key enhancements to the manuscript:

- **Improved Visualization (Reviewer 1):** In line with Reviewer 1's recommendation regarding categorical color scales, we have completely updated **Figure 3B** using a technical solution that randomizes maximally distinct colors. This significantly improves the visual contrast between individual plasmid species trajectories on the Muller plot.
- **Extensive Robustness Analyses (Reviewer 2):** To address Reviewer 2's main query regarding the potential impact of different vertical transmission (VT) and horizontal transfer (HT) rate combinations, we have performed new sensitivity analyses. We now provide **two new figures in the Supplementary Information (Figs. S4 and S6)**. These results explicitly demonstrate that drastically increasing VT efficiency while lowering HT rates does not qualitatively change our findings or our core conclusions.
- **Minor Revisions:** We have carefully addressed all other minor and technical suggestions raised by Reviewer 2 to further polish the manuscript.

Below, we provide a detailed, point-by-point response to each of the reviewers' comments.

Reviewer #1:

[identifies himself as Bram van Dijk]

Dear authors,

I am happy with the revisions, and happy to see this work published. I did however submit 'minor revisions' as I still would like one of my suggestions to be followed through (but it remains a suggestion). I would still consider is changing the colour scale of 3B from viridis to randomised colours. There are plenty of packages available to do this. There is no order in the species data, so assigning colours along a continuous scale is really a bad choice in my opinion.

R1.1: We thank the reviewer for their positive evaluation and for emphasizing this visualization point. We agree that since plasmid species lack a natural ordering, a continuous color scale like *viridis* could imply a non-existent gradient. Likewise, we have successfully implemented your suggestion using the `distinctipy` Python package. We use the function *distinctipy.get_colors(200)* to obtain the 200 colors as distinct as possible. Then, we randomly shuffle these 200 colors so that there is no longer any order to the plasmid species represented on the Muller-plot.

As shown in the updated **Fig. 3B** (reproduced below), this technical solution significantly improves the visual contrast between individual plasmid species trajectories.

The updated code to obtain the new figure is still available on the Zenodo repository (file *figure_3_neutral_model.ipynb*).

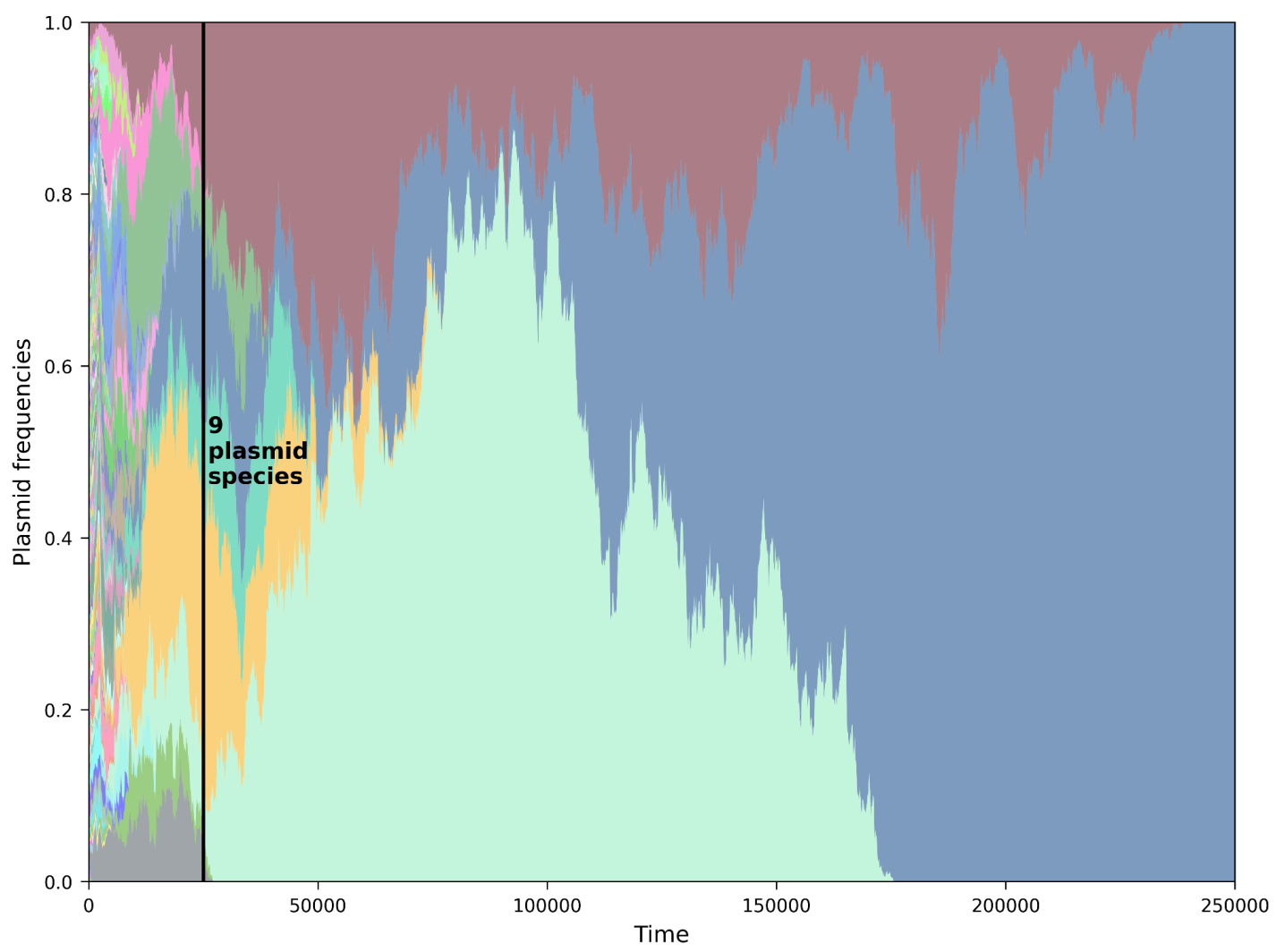

Updated Figure 3B: Ecological drift in action. Without dispersal or innovation, stochasticity alone shapes diversity. (Upper row) Plasmid species richness declines over time as species go extinct. (Lower row) Frequencies of plasmid species fluctuate stochastically. After the early loss of many species, reaching complete exclusion (one species remaining) can take a long time.

Reviewer #2:

The authors have responded satisfactorily to all my suggestions and comments, and I'm very glad to see the deeper analyses controlling for potential outbreak clones — this is very nice.

However, now that they have provided full details about model parameterisation I'm afraid to say have a new query that I'd like reassurance on:

- The authors state that their vertical transmission rate was set to 0.9, meaning there's a 10% chance of losing the plasmid each generation. This seems high when considering the literature and e.g. values in the provided citation, particularly when considering most natural transmissible plasmids have partitioning apparatus and toxin-antitoxin systems. The Lau et al. 2013 paper suggests that rates of loss are often as low as 1e-6. Are the main findings robust to higher vertical transmission rates?

- Similarly, the use of horizontal transmission rates between 1e-5 and 1e-7 is at the higher end of those indicated in the provided references, which indicate plasmids frequently have transmission rates 1e-14 to 1e-10. How robust are the results to (much) lower horizontal transfer rates?

I don't think the values chosen by the authors are unreasonable, and I appreciate it can be hard to match up real-world measurements with abstract model formulations. It would be good to know, though, that the authors observations do not just emerge from a peculiar regime of highly transmissible, easily-lost plasmids.

R2.1: We thank the reviewer for this pertinent comment regarding the sensitivity of our model to vertical transmission (VT) and horizontal transfer rates (HTR). We agree that exploring how these parameters impact the robustness of our results is important.

Below, we first address the scaling differences between simulated and empirical rates, explain the biological necessity of our baseline parameter choices, and introduce two new supplementary figures demonstrating that our core findings remain highly robust to change in VT and HTR.

1. Scaling Considerations for Horizontal Transfer Rates

In our study, computational constraints limit our stochastic simulations to bacterial populations of 1e6 cells (simulating larger populations would lead to prohibitive computation times). A population of 1e6 cells is roughly equivalent to the density found in a tiny 1µL volume of a standard liquid microbial culture.

Consequently, in our model, the dimensions of the horizontal transfer rates (α) are expressed in $\mu L.\, cells^{-1}.\, t^{-1}$, whereas experimental literature quantifies them in $mL.\, cells^{-1}.\, h^{-1}$. To compare our simulated rates with real-world measurements, we must rescale them (assuming approximately 3 replications occur per hour). For instance, our baseline rate of $\alpha = 1e - 5$ in the neutral model translates to an empirical rate of approximately $3e - 8\, mL.\, cells^{-1}.\, h^{-1}$ which aligns with some reported values for active natural plasmids.

2. Parameter Combinations for Plasmid Persistence

To study plasmid community assembly and competition for a shared host resource, each plasmid species must fundamentally be capable of persisting on its own. If a plasmid goes extinct when

alone, its disappearance is due to an intrinsic lack of viability rather than the competitive structural forces we aim to evaluate.

The mathematical condition for a single plasmid species *i* to invade and persist when rare can be derived analytically from our deterministic model. The growth rate of a rare plasmid species *i* introduced into a plasmid-free population at equilibrium ($\widehat{B_0} = \frac{r_0 - c}{d}$) is given by:

$$\frac{B_i'}{B_i}_{\{\widehat{B_0},0,\ldots,0\}} = r_i\varepsilon_i - c - d\widehat{B_0} + \alpha_i\widehat{B_0}$$

Imposing this invasion condition yields the precise threshold value for the horizontal transfer rate α_i required for persistence:

$$\alpha_i > \frac{d(r_0 - r_i\varepsilon_i)}{r_0 - c}$$

With our baseline parameters ($d = 1e - 6$, $r_0 = 1$, $r_i = 0.99$, and a vertical transmission probability of $\varepsilon_i = 0.9$, the minimum HTR required to withstand environmental stress ($c = 0.9$) is $\alpha_i = 1.1e - 6$.

However, as the reviewer rightly notes, if we drastically increase the vertical transmission efficiency to simulate highly stable partitioning or toxin-antitoxin systems (e.g. $\varepsilon_i = 0.999999$), the analytical invasion threshold drops significantly, allowing the plasmid to persist at a much lower horizontal transfer rate ($\alpha_i \geq 1.0001e - 7$, equivalent to $3e - 10\, mL.cells^{-1}.h^{-1}$).

3. Manuscript Modifications and New Robustness Analyses

To explicitly address the reviewer's query and demonstrate that our conclusions are not tied to a peculiar parameter regime, we have performed new sensitivity analyses and integrated two new figures in the Supplementary Information (Figs. S4 and S6) considering a high probability of vertical transfer.

- **In the Neutral Model (New Fig. S4):** Lau et al. (2013) report plasmid loss rates ranging from 1e-3 (for mini-R1) to as low as 1e-6 (for pSC101+hok/sok). We re-ran our neutral model by setting the vertical transmission probability to $\varepsilon = 0.999$ or $\varepsilon = 0.999999$ (equivalent to loss rate of1e-3 and 1e-6 respectively), allowing us to test much lower HTR ($\alpha = 1.01e - 7\, \mu L.cells^{-1}.t^{-1}$and $\alpha = 1.0001e - 7\, \mu L.cells^{-1}.t^{-1}$ respectively, equivalent to around $3e - 10\, mL.cells^{-1}.h^{-1}$). The resulting Rank-Abundance Curves are qualitatively identical to those presented in the main text (Fig. S4). We now refer to this robustness check in the main text (l.271-273).

We have also updated our parameter table to explicitly highlight this scaling relationship (Table S2).

- **In the Unified Model (New Fig. S6):** In this model, the problem is dynamically different because recombination rates evolve freely, and interconnected populations face highly

contrasted environmental stress regimes. While a purely analytical boundary cannot be mapped here, we re-ran the evolutionary simulations fixing the vertical transmission rate to a highly stable level ($\varepsilon_{min} = 0.999$ and $\varepsilon_{max} = 0.999999$) across a broader range of HTR ($\alpha_{min} = 1e - 15$ to $\alpha_{max} = 1e - 5$). As shown in Figure S6, the core finding remains entirely robust: the same three functional plasmid clusters emerge. Because vertical transmission is nearly perfect, plasmid loss is rare, leading to fewer plasmid-free cells and fewer total horizontal transfer events. Consequently, evolutionary convergence takes longer, requiring extended simulation trajectories ($T = 5,000$), but the qualitative outcome remains unchanged. This is now proposed in the main text (l. 352-357).

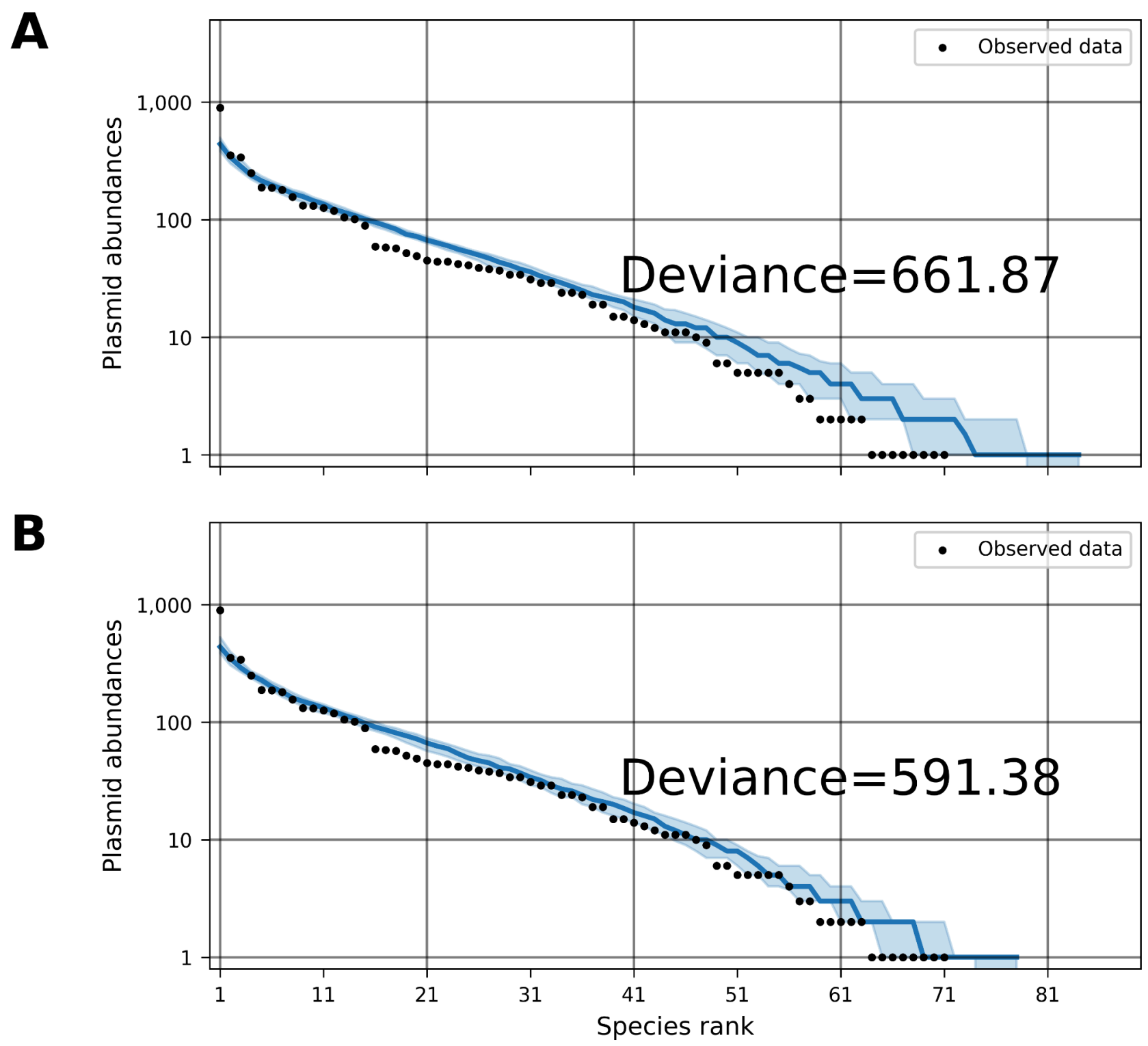

Figure S4: Neutral model with low horizontal transfer rate and high vertical transfer probability. Observed plasmid diversity (black dots) is compared with simulations. The median (blue curve) and interquartile (light blue area) densities for each rank are represented from 25 simulations. The mean deviance between observed and simulated data is computed (all others parameters are as defined in table S3 and are the same as in Fig. 3 in the main text). **(A) When** $\alpha_i = 1.0001e - 7, \forall i \in \{1,..., 200\}$ **and** $\varepsilon_i = 0.999999, \forall i \in \{1,..., 200\}$**. (B) When** $\alpha_i = 1.01e - 7, \forall i \in \{1,..., 200\}$ **and** $\varepsilon_i = 0.999, \forall i \in \{1,..., 200\}$**.**

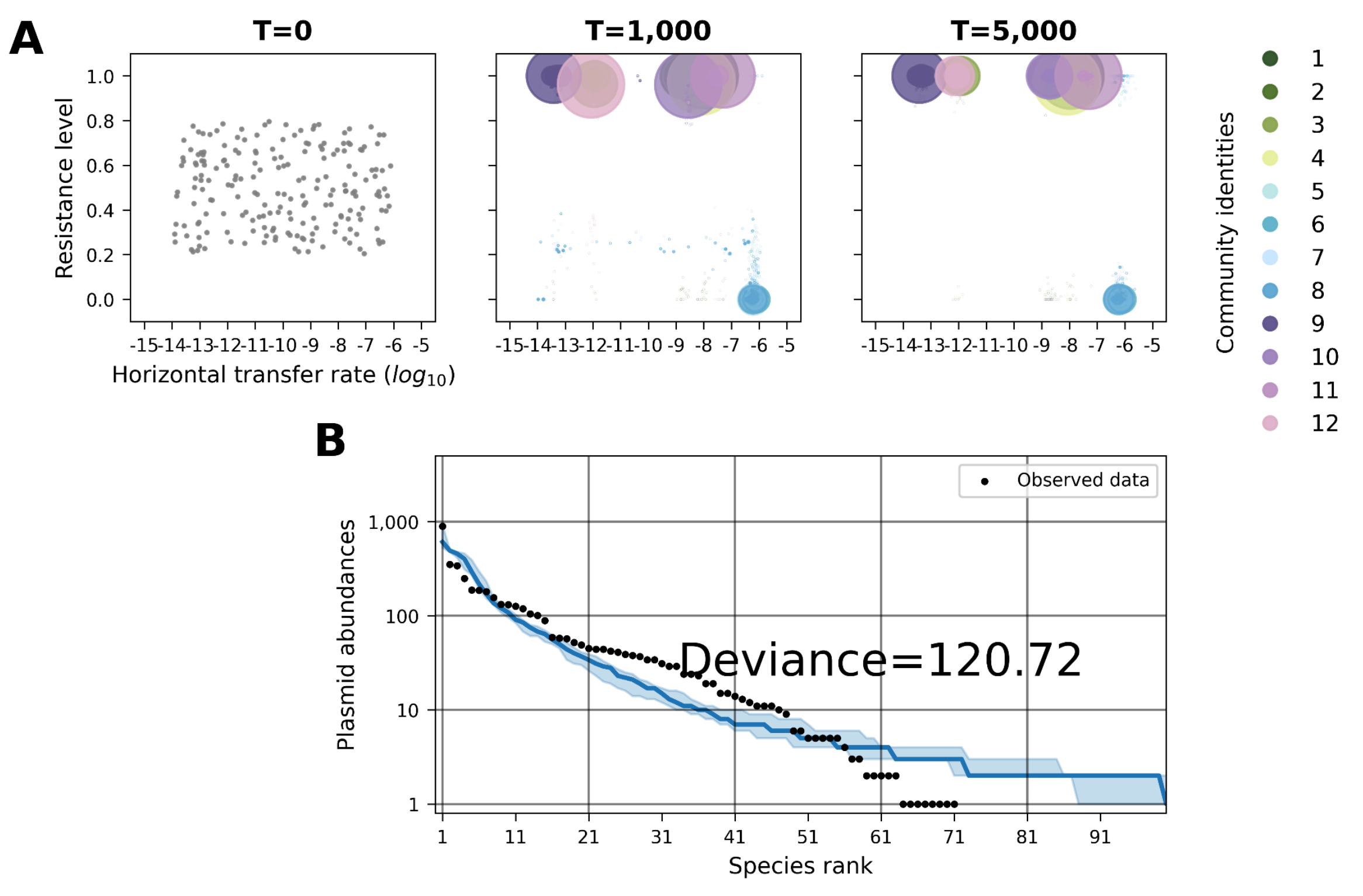

Figure S6: Unified model with high vertical transfer probability ($\varepsilon_{min} = 0.999$, $\varepsilon_{max} = 0.999999$) and low horizontal transfer rate ($\alpha_{min} = 1e-15$, $\alpha_{max} = 1e-5$). A. Distribution of plasmid densities in the space of plasmid traits. Each dot represents a plasmid species in trait space (HTR on the x-axis, RL on the y-axis). Dot size reflects abundance; color indicates the community identity. (T=0) Initial species traits are randomly distributed with equal abundances. (T=1,000) After 1,000 generations, plasmids begin diverging toward peripheral strategies, high/low resistance, high/low transfer, or both. (T=5,000) After 5,000 generations, three distinct clusters have emerged: (i) high RL with low HTR, (ii) high HTR with low RL, and (iii) high HTR with high RL. This reflects an eco-evolutionary sorting driven by local stress regimes and connectivity across communities. **B. Observed plasmid diversity (black dots) is compared with simulations.** The median (blue curve) and interquartile (light blue area) densities for each rank are represented from 25 simulations. The mean deviance between observed and simulated data is computed.

<u>Minor comments/issues:</u>

- Methods section. Section on plasmid diversity could be clarified (line 502): "We filtered all complete E. coli, Klebsiella, and Salmonella genomes available on NCBI RefSeq…". Later in the paragraph (line 507): "We extracted a total of 6,131 (E. coli), 5,149 (Klebsiella) and 1,517 (Salmonella) plasmids for downstream analysis." Similar later in the paragraph (line 510)

R2.2: We clarified the section on plasmid diversity as follows (l.513-525):

"To mitigate the risk of biased sampling and the over-representation of pandemic clones commonly found in public databases, we implemented a stringent genomic de-replication step. We filtered all complete *E. Coli, Klebsiella* and *Salmonella* genomes available on NCBI RefSeq (last accessed March

2023) using a sequence-based approach to exclude closely related strains. By applying a MASH distance threshold of <0.0001, we ensured that our dataset consists of non-redundant, genomically independent hosts, thereby avoiding the inclusion of pseudo-replicates from clonal outbreaks. From this de-replicated set, we extracted a total of 6,131 (*E. Coli*), 5,149 (*Klebsiella*) and 1,517 (*Salmonella*) plasmids for downstream analysis. We then clustered these plasmids in plasmid taxonomic units or PTU using COPLA v1.0 and default parameters ((121)). We obtained 71 (E. Coli), 61 (*Klebsiella*) and 47 (*Salmonella*) PTU containing a total of 4,411 (*E. Coli*), 3,277 (*Klebsiella*) and 1,168 (*Salmonella*) plasmids. The remaining plasmids were not assigned to known PTUs. We then could count the number of plasmids for each PTU. Data available in PTU_count.tsv (and plasmid_data_sup.xlsx for *Klebsiella* and *Salmonella* genera)."

- Overlapping labels in Figure 1.

R2.3: Indeed, x-labels on Fig. 1D were overlapped. This is now corrected.

- Figure 3C guide does not match what's described in the legend.

R2.4: We agree that guides on FIG. 3C did not match the legend description. We completed these guides (adding "drift" where necessary) so they match the legend description.

- RAC abbreviation introduced more than once (line 133, 355, 410) — this isn't necessarily a problem though, it's often nice to be reminded what acronyms stand for!

R2.5: Indeed the same RAC abbreviation was introduced multiple times in the paper. We now introduce the abbreviation once (on l.113) and then use this abbreviation or the complete expression.

- Line 960 typo (Escherichai); line 965 (Kelbsiella)

R2.6: Thanks! These typos were corrected.

- Line 971. Is text missing from this sentence (e.g. between the brackets, at the end of the sentences)? It appears correctly in the .docx but not in the .pdf. Also on line 975.

R2.7: Yes, equations were missing. We fixed this.

- Total bacterial density parameter not present in the table S2. Is this an error?

R2.8: Yes, parameters were missing in this table. It was corrected.

Reviewer #3:

The authors have substantially improved the manuscript and have clarified the scope of the contribution. The revised version no longer presents the ecological analogy itself as the main novelty. Instead, it more clearly focuses on plasmid assemblage diversity and on the quantitative fit between ecological models and plasmid rank-abundance patterns. The strongest biological contribution is the proposed community-assembly framework for interpreting plasmid pangenomes. I also appreciate the authors' effort to identify a more concrete biological implication of the model: that strong antibiotic pressure may accelerate competitive exclusion and reduce the diversity of rare plasmids. However, I do not view this as a fundamentally new prediction, since it follows directly from standard ecological expectations under strong directional selection. Its value lies more in connecting this expectation to plasmid pangenome data than in proposing a new mechanism.

Overall, the manuscript is clearer and stronger than the previous version. Nevertheless, I still view its novelty as moderate, since the main contribution lies in integrating and applying existing ecological concepts to plasmid assemblages rather than in developing a fundamentally new biological principle.

R3.1: We thank the reviewer for their positive evaluation of our revisions and for recognizing that our quantitative fit of rank-abundance patterns and the community-assembly framework constitute a strong biological contribution to interpreting plasmid pangenomes.

We agree with the reviewer that our model applies established ecological principles rather than inventing new mathematical laws of ecology. However, we respectfully emphasize that transferring these concepts into a fully quantified, predictive framework for mobile genetic elements (MGEs) represents a major conceptual shift for microbial genomics.

As the reviewer notes, the true value of this work lies in connecting theoretical expectations with empirical pangenome data. By demonstrating that the massive data generated by modern sequencing can be structurally explained by niche and neutral processes, we hope this paper will encourage plasmid biologists and genomicists to realize that ecology offers a robust theoretical and methodological foundation for their systems. We view this study as a foundational step toward developing a true "ecology of the bacterial genome," where much remains to be built, and we are grateful to the reviewer for helping us sharpen this message.
