# Supplementary material for: Ecological theory sheds light on plasmid diversity and dynamics
Source: PLoS Biol. 2026 Aug 28;24(8):e3003918. doi: 10.1371/journal.pbio.3003918 (PMC13524261; doi:10.1371/journal.pbio.3003918)
Supplement: Supplementary file 3 [file pbio.3003918.s007.doc]

| **Data** | **Fitted model** | **Number of parameters** | **AIC** |
| --- | --- | --- | --- |
| ***E. coli* plasmids (Fig 1A)** | Broken-stick | 0 | 731.74 |
|  | Fisher’s log-series | 1 | 685.64 |
|  | Geometric | 1 | 729.19 |
|  | Poisson-lognormal | 2 | 686.91 |
|  | Lognormal | 2 | 689.98 |
|  | Pueyo’s power-bend | 2 | 682.99 |
| **Birds (Fig 1B)** | Broken-stick | 0 | 2519.75 |
|  | Fisher’s log-series | 1 | 2324.17 |
|  | Geometric | 1 | 2504.60 |
|  | Poisson-lognormal | 2 | 2343.64 |
|  | Lognormal | 2 | 2376.50 |
|  | Pueyo’s power-bend | 2 | 2323.83 |
| **Trees (Fig 1C)** | Broken-stick | 0 | 2505.06 |
|  | Fisher’s log-series | 1 | 2322.90 |
|  | Geometric | 1 | 2500.60 |
|  | Poisson-lognormal | 2 | 2309.94 |
|  | Lognormal | 2 | 2318.03 |
|  | Pueyo’s power-bend | 2 | 2310.55 |
| ***Klebsiella* plasmids (S1A Fig.)** | Broken-stick | 0 | 611.55 |
|  | Fisher’s log-series | 1 | 568.03 |
|  | Geometric | 1 | 608.88 |
|  | Poisson-lognormal | 2 | 569.06 |
|  | Lognormal | 2 | 572.17 |
|  | Pueyo’s power-bend | 2 | 567.30 |
| ***Salmonella* plasmids (S1B Fig.)** | Broken-stick | 0 | 397.70 |
|  | Fisher’s log-series | 1 | 377.48 |
|  | Geometric | 1 | 396.10 |
|  | Poisson-lognormal | 2 | 379.09 |
|  | Lognormal | 2 | 381.80 |
|  | Pueyo’s power-bend | 2 | 377.40 |
