# Supplementary material for: Ecological theory sheds light on plasmid diversity and dynamics
Source: PLoS Biol. 2026 Aug 28;24(8):e3003918. doi: 10.1371/journal.pbio.3003918 (PMC13524261; doi:10.1371/journal.pbio.3003918)
Supplement: Supplementary file 4 [file pbio.3003918.s008.doc]

| **Variables (unit)** | **Definition** | **Values in Fig 2 and S2A-B Fig.** | **Values in S2C Fig.** | **Values in S2D Fig.** | |  |  |  |  |  |
| --- | --- | --- | --- | --- | --- | --- | --- | --- | --- | --- |
| $B_{0}$($cells.\mu L^{-1}$) | Density of plasmid-free bacteria | $B_{0}\left( 0 \right)={1e6}/3$ | $B_{0}\left( 0 \right)=1e5$ | $B_{0}\left( 0 \right)=1e5$ | |  |  |  |  |  |
| $n$ | Number of plasmid species | $n=2$ | $n=200$ | $n=200$ | |  |  |  |  |  |
| $B_{i}$ for $i=1,...,n$ ($cells.\mu L^{-1}$) | Density of plasmid-*i*-carrying bacteria | $B_{res}\left( 0 \right)={1e6}/3$ when present and $B_{sens}\left( 0 \right)={1e6}/3$ when present | $B_{i}\left( 0 \right)=10$  $\forall i\in\left\{ 1,...,200 \right\}$ | $B_{i}\left( 0 \right)=10$  $\forall i\in\left\{ 1,...,200 \right\}$ | |  |  |  |  |  |
| $B=B_{0}+\sum B_{i}$($cells.\mu L^{-1}$) | Total bacterial density |  |  |  | |  |  |  |  |  |
| **Parameters (unit)** |  |  |  |  | **Justification** | | | | | |
| $r_{0}$($t^{-1}$) | *Per capita* replication rate of plasmid-free bacteria | 1 | 1 | 1 | We take the replication rate of plasmid-free bacteria equal to 1 so that in the absence of competition and stress, time is a generation time, meaning that the bacterial population doubles with each unit of time. | | | | | |
| $d$ ($\mu L.{cells}^{-1}.t^{-1}$) | Competition coefficient *per capita* | $1e-6$ | $1e-6$ | $1e-6$ | With $r_{0}=1$, we take $d=1e-6$, so that maximal bacterial carrying capacity is ${r_{0}}/d=1e6$ $cells.\mu L^{-1}$consistent with stationary-phase batch cultures reported in the literature [(](https://www.zotero.org/google-docs/?XKCnph)1[)](https://www.zotero.org/google-docs/?XKCnph). | | | | | |
| $r_{i}$ for $i=1,...,200$ ($t^{-1}$) | *Per capita* replication rate of plasmid-*i*-carrying bacteria | $r_{res}=0.9$ and $r_{sens}=0.99$ | $r_{min}=0.9$ and $r_{max}=0.99$ | $r_{i}=0.99$  $\forall i\in\left\{ 1,...,200 \right\}$ | We take the replication rate of a sensitive (resistant respectively) plasmid-carrying bacteria $r_{sens}=0.99$ ($r_{res}=0.9$), meaning that a sensitive (resistant) plasmid reduces its bacterial host fitness by 1% (10%), consistent with some observed values [(26)](https://www.zotero.org/google-docs/?fusu21). | | | | | |
| $\epsilon_{i}$ for $i=1,...,200$ (dimensionless) | *Per capita* probability of vertical transfer of plasmid *i* to daughter cell during bacterial replication | $\epsilon_{res}=0.95$ and $\epsilon_{sens}=0.9$ | $\epsilon_{i}=0.95$ $\forall i\in\left\{ 1,...,200 \right\}$ | $\epsilon_{min}=0.9$ and $\epsilon_{max}\approx0.925$ | We consider the probability of vertical transfer of a plasmid between 0.9 and 0.95, meaning that at worst, a plasmid is stochastically lost once every 10 generations, consistent with some observations [(2)](https://www.zotero.org/google-docs/?C8ca78). | | | | | |
| $s_{i}$ for $i=1,...,200$ (dimensionless) | Antibiotic stress mortality resistance level induced by plasmid *i* | $s_{res}=1$ and $s_{sens}=0$ | $s_{min}=0$ and $s_{max}=1$ | $s_{i}=0$  $\forall i\in\left\{ 1,...,200 \right\}$ | We consider that a resistant plasmid confers full resistance to the antibiotic stress, whereas a sensible plasmid confers no resistance at all. | | | | | |
| $\alpha_{i}$ for $i=1,...,200$ ($\mu L.{cells}^{-1}.t^{-1}$) | *Per capita²* horizontal transfer rate of plasmid *i* | $\alpha_{res}=1e-6$ and $\alpha_{sens}=1e-5$ | $\alpha_{i}=1e-6$ $\forall i\in\left\{ 1,...,200 \right\}$ | $\alpha_{min}=1e-7$and $\alpha_{max}=1e-5$ | We consider the plasmid horizontal transfer rate between $1e-7\mu L.{cells}^{-1}.t^{-1}$ and $1e-5\mu L.{cells}^{-1}.t^{-1}$ corresponding to transfer rate between$3e-10mL.{cells}^{-1}.h^{-1}$ and $3e-8mL.{cells}^{-1}.h^{-1}$consistent with observations [(38,](https://www.zotero.org/google-docs/?r65VEm)41[)](https://www.zotero.org/google-docs/?r65VEm). | | | | | |

**Reference**

1. [Kumsuwan, S. *et al.* (2025) ‘A bayesian approach for parameterizing and predicting plasmid conjugation dynamics’, *Scientific Reports*, 15(1), p. 7396. Available at: https://doi.org/10.1038/s41598-024-82799-5.](https://www.zotero.org/google-docs/?XFEvl4)

2. [Lau, B.T.C., Malkus, P. and Paulsson, J. (2013) ‘New quantitative methods for measuring plasmid loss rates reveal unexpected stability’, *Plasmid*, 70(3), pp. 353–361. Available at: https://doi.org/10.1016/j.plasmid.2013.07.007.](https://www.zotero.org/google-docs/?XFEvl4)
