# Supplementary material for: Ecological theory sheds light on plasmid diversity and dynamics
Source: PLoS Biol. 2026 Aug 28;24(8):e3003918. doi: 10.1371/journal.pbio.3003918 (PMC13524261; doi:10.1371/journal.pbio.3003918)
Supplement: Supplementary file 5 [file pbio.3003918.s009.doc]

| **Variables (unit)** | **Definition** | **Values in Fig 3B** | **Values in Fig 3C-E** | **Values in S3 Fig.** | |  |  |  |  |  |
| --- | --- | --- | --- | --- | --- | --- | --- | --- | --- | --- |
| $B_{0}$ ($cells.\mu L^{-1}$) | Density of plasmid-free bacteria | $B_{0}\left( 0 \right)=1e5$ | $B_{0}\left( 0 \right)=1e5$ | $B_{0}\left( 0 \right)=100$ | |  |  |  |  |  |
| $n$ | Number of plasmid species | $n=200$ | $n=200$ | $n=200$ | |  |  |  |  |  |
| $m$ | Number of community | $m=1$ | $m=4$ | $m=4$ | |  |  |  |  |  |
| $\Delta_{t}$($t$) | Time step | $\Delta_{t}=0.1$ | $\Delta_{t}=0.1$ | $\Delta_{t}=0.1$ | |  |  |  |  |  |
| $T$ | Final time | $T=250,000$ | $T=25,000$ | $T=25,000$ | |  |  |  |  |  |
| $B_{i}$for $i=1,...,200$  ($cells.\mu L^{-1}$) | Density of plasmid-*i*-carrying bacteria | $B_{i}\left( 0 \right)=10$ $\forall i\in\left\{ 1,...,200 \right\}$ | $B_{i}\left( 0 \right)=10$ $\forall i\in\left\{ 1,...,200 \right\}$ | $B_{i}\left( 0 \right)=10$ $\forall i\in\left\{ 1,...,200 \right\}$ | |  |  |  |  |  |
| $B=B_{0}+\sum B_{i}$ ($cells.\mu L^{-1}$) | Total bacterial density |  |  |  | |  |  |  |  |  |
| **Parameters (unit)** |  |  |  |  | **Justification** | | | | | |
| $r_{0}$ ($t^{-1}$) | *Per capita* replication rate of plasmid-free bacteria | 1 | 1 | 1 | Same justification as S2 Tab. | | | | | |
| $d$ ($\mu L.{cells}^{-1}.t^{-1}$) | Competition coefficient p*er capita* | $1e-6$ | $1e-6$ | $1e-6$ | Same justification as S2 Tab. | | | | | |
| $r_{i}$ for $i=1,...,200$ ($t^{-1}$) | *Per capita* replication rate of plasmid-*i*-carrying bacteria | $r_{i}=0.99$ $\forall i\in\left\{ 1,...,200 \right\}$ | $r_{i}=0.99$ $\forall i\in\left\{ 1,...,200 \right\}$ | $r_{i}=0.99$ $\forall i\in\left\{ 1,...,200 \right\}$ | To reflect a very low cost of plasmid, corresponding to 1%, we set $r_{i}=0.99$ | | | | | |
| $\epsilon_{i}$ for $i=1,...,200$ (dimensionless) | *Per capita* probability of vertical transfer of plasmid *i* to daughter cell during bacterial replication | $\epsilon_{i}=0.9$ $\forall i\in\left\{ 1,...,200 \right\}$ | $\epsilon_{i}=0.9$ $\forall i\in\left\{ 1,...,200 \right\}$ | $\epsilon_{i}=0.9$ $\forall i\in\left\{ 1,...,200 \right\}$ | To reflect a low probabiblity of plasmid loss, we set $\epsilon_{i}=0.9$ | | | | | |
| $s_{i}$ for $i=1,...,200$ (dimensionless) | Antibiotic stress mortality resistance level induced by plasmid *i* | $s_{i}=0$ $\forall i\in\left\{ 1,...,200 \right\}$ | $s_{i}=0$ $\forall i\in\left\{ 1,...,200 \right\}$ | $s_{i}=0$ $\forall i\in\left\{ 1,...,200 \right\}$ | No AMR genes. | | | | | |
| $\alpha_{i}$ for $i=1,...,200$ ($\mu L.{cells}^{-1}.t^{-1}$) | *Per capita²* horizontal transfer rate of plasmid *i* | $\alpha_{i}=1e-5$ $\forall i\in\left\{ 1,...,200 \right\}$ | $\alpha_{i}=1e-5$ $\forall i\in\left\{ 1,...,200 \right\}$ | $\alpha_{i}=1e-5$ $\forall i\in\left\{ 1,...,200 \right\}$ | We consider plasmids with high HTR [(](https://www.zotero.org/google-docs/?if9E56)38,41[)](https://www.zotero.org/google-docs/?if9E56). | | | | | |
| $\tau$ ($t^{-1}$) | Innovation rate | $\tau=0$ | $\tau=0$ or $\tau=1.25e-5$ | $\tau\in\left\{ 0,1e-6,1e-5,1.25e-5,2.5e-5,5e-5,1e-4 \right\}$ | Sensitivity analysis | | | | | |
| $\Gamma$ ($t^{-1}$) | Dispersal rate | $\Gamma=0$ | $\Gamma=0$ or $\Gamma=1e-3$ | $\Gamma\in\left\{ 0,1e-6,1e-5,1e-4,1e-3,1e-2,1e-1 \right\}$ | Sensitivity analysis | | | | | |
