# Supplementary material for: Ecological theory sheds light on plasmid diversity and dynamics
Source: PLoS Biol. 2026 Aug 28;24(8):e3003918. doi: 10.1371/journal.pbio.3003918 (PMC13524261; doi:10.1371/journal.pbio.3003918)
Supplement: Supplementary file 6 [file pbio.3003918.s010.doc]

| **Variables (unit)** | **Definition** | **Values in Fig 4 and S4A Fig.** | **Values in S4B-D Fig.** | |  |  |  |  |
| --- | --- | --- | --- | --- | --- | --- | --- | --- |
| $B_{0}$ ($cells.\mu L^{-1}$) | Density of plasmid-free bacteria | $B_{0}\left( 0 \right)=1e3$ | $B_{0}\left( 0 \right)=100$ | |  |  |  |  |
| $n$ | Number of plasmid species | $n=200$ | $n=200$ | |  |  |  |  |
| $m$ | Number of community | $m=12$ | $m=12$ | |  |  |  |  |
| $\Delta_{t}$($t$) | Time step | $\Delta_{t}=0.1$ | $\Delta_{t}=0.1$ | |  |  |  |  |
| $T$ | Final time | $T=1,000$ | $T=1,000$ | |  |  |  |  |
| $B_{i}$for $i=1,...,200$  ($cells.\mu L^{-1}$) | Density of plasmid-*i*-carrying bacteria | $B_{i}\left( 0 \right)=10$ $\forall i\in\left\{ 1,...,200 \right\}$ | $B_{i}\left( 0 \right)=10$ $\forall i\in\left\{ 1,...,200 \right\}$ | |  |  |  |  |
| $B=B_{0}+\sum B_{i}$ ($cells.\mu L^{-1}$) | Total bacterial density |  |  | |  |  |  |  |
| **Parameters (unit)** |  |  |  | **Justification** | | | | |
| $r_{0}$ ($t^{-1}$) | *Per capita* replication rate of plasmid-free bacteria | 1 | 1 | Same justification as S2 Tab. | | | | |
| $d$ ($\mu L.{cells}^{-1}.t^{-1}$) | Competition coefficient p*er capita* | $1e-6$ | $1e-6$ | Same justification as S2 Tab. | | | | |
| $r_{i}$ for $i=1,...,200$ ($t^{-1}$) | *Per capita* replication rate of plasmid-*i*-carrying bacteria | $r_{min}=0.9$ and $r_{max}=0.99$ | $r_{min}=0.9$ and $r_{max}=0.99$ | Same justification as S2 Tab. | | | | |
| $\epsilon_{i}$ for $i=1,...,200$ (dimensionless) | *Per capita* probability of vertical transfer of plasmid *i* to daughter cell during bacterial replication | $\epsilon_{min}=0.9$ and $\epsilon_{max}=0.99$ | $\epsilon_{min}=0.9$ And $\epsilon_{max}=0.99$ | Same justification as S2 Tab. | | | | |
| $s_{i}$ for $i=1,...,200$ | Antibiotic stress mortality resistance level induced by plasmid *i* | Initially, $s_{i}\sim Unif\left( 0.2,0.8 \right)$ | Initially, $s_{i}\sim Unif\left( 0.2,0.8 \right)$ | This corresponds to plasmids initially conferring a 20-80% resistance to antibiotic stress. | | | | |
| $\alpha_{i}$ for $i=1,...,200$ | *Per capita²* horizontal transfer rate of plasmid *i* | Initially, $\alpha_{i}\sim1e-Unif\left( 11,6 \right)$ | Initially,  $\alpha_{i}\sim1e-Unif\left( 11,6 \right)$ | This corresponds to plasmid initially having a horizontal transfer rate ranging from to . | | | | |
| $\tau$ | Innovation rate | $\tau=1e-4$ | $\tau\in\left\{ 0,1e-6,1e-5,1.25e-5,2.5e-5,5e-5,1e-4 \right\}$ | Sensitivity analysis | | | | |
| $\Gamma$ | Dispersal rate | $\Gamma=1e-9$ | $\Gamma\in\left\{ 0,1e-9,1e-6,1e-5,1e-4,1e-3,1e-2 \right\}$ | Sensitivity analysis | | | | |
