# Supplementary material for: Ecological theory sheds light on plasmid diversity and dynamics
Source: PLoS Biol. 2026 Aug 28;24(8):e3003918. doi: 10.1371/journal.pbio.3003918 (PMC13524261; doi:10.1371/journal.pbio.3003918)
Supplement: Peer review history

# Editor report: Decision Letter 0

**DOI:** 10.1371/journal.pbio.3003918.r001

**Authors:** Roland Roberts

19 Nov 2025

Dear Dr Tuffet,

Thank you for submitting your manuscript entitled "Plasmid diversity and dynamics in the light of ecological theories" for consideration as a Research Article by PLOS Biology.

Your manuscript has now been evaluated by the PLOS Biology editorial staff, as well as by an academic editor with relevant expertise, and I’m writing to let you know that we would like to send your submission out for external peer review.

IMPORTANT: We think that your paper would be best considered as a Short Report. No re-formatting is required, but please select"Short Reports" as the article type when you upload your additional metadata (see next paragraph).

However, before we can send your manuscript to reviewers, we need you to complete your submission by providing the metadata that is required for full assessment. To this end, please login to Editorial Manager where you will find the paper in the 'Submissions Needing Revisions' folder on your homepage. Please click 'Revise Submission' from the Action Links and complete all additional questions in the submission questionnaire.

Once your full submission is complete, your paper will undergo a series of checks in preparation for peer review. After your manuscript has passed the checks it will be sent out for review. To provide the metadata for your submission, please Login to Editorial Manager (https://www.editorialmanager.com/pbiology) within two working days, i.e. by Nov 21 2025 11:59PM.

If your manuscript has been previously peer-reviewed at another journal, PLOS Biology is willing to work with those reviews in order to avoid re-starting the process. Submission of the previous reviews is entirely optional and our ability to use them effectively will depend on the willingness of the previous journal to confirm the content of the reports and share the reviewer identities. Please note that we reserve the right to invite additional reviewers if we consider that additional/independent reviewers are needed, although we aim to avoid this as far as possible. In our experience, working with previous reviews does save time.

If you would like us to consider previous reviewer reports, please edit your cover letter to let us know and include the name of the journal where the work was previously considered and the manuscript ID it was given. In addition, please upload a response to the reviews as a 'Prior Peer Review' file type, which should include the reports in full and a point-by-point reply detailing how you have or plan to address the reviewers' concerns.

During the process of completing your manuscript submission, you will be invited to opt-in to posting your pre-review manuscript as a bioRxiv preprint. Visit http://journals.plos.org/plosbiology/s/preprints for full details. If you consent to posting your current manuscript as a preprint, please upload a single Preprint PDF.

Feel free to email us at plosbiology@plos.org if you have any queries relating to your submission.

Kind regards,

Roli Roberts

Roland Roberts, PhD

Senior Editor

PLOS Biology

rroberts@plos.org

# Peer review: Decision Letter 1

**DOI:** 10.1371/journal.pbio.3003918.r002

**Authors:** Roland Roberts

23 Jan 2026

Dear Dr Tuffet,

Thank you for your patience while your manuscript "Plasmid diversity and dynamics in the light of ecological theories" was peer-reviewed at PLOS Biology. It has now been evaluated by the PLOS Biology editors, an Academic Editor with relevant expertise, and by three independent reviewers.

You'll see that reviewer #1 is very positive, but suggests some re-framing of the middle of the paper, mentions a preprint, and has a list of mostly presentational suggestions. Reviewer #2 thinks that despite a degree of obviousness, the study has the potential to have impact; however, s/he has some concerns about possible biases in the data, wants more clarity around parameterisation of the model, and some formal stats tests. Reviewer #3 is rather more negative, flagging a number of papers that have explored similar perspectives, and which mean that the current paper falls short of offering conceptual novelty.

We discussed the reviewers' comments with the Academic Editor, and we think that although you should respond in full to reviewer #3's concerns, overall we think that after appropriate revision, your study is appropriate for further consideration at PLOS Biology. In light of the reviews, which you will find at the end of this email, we would therefore like to invite you to revise the work to thoroughly address the reviewers' reports.

Given the extent of revision needed, we cannot make a decision about publication until we have seen the revised manuscript and your response to the reviewers' comments. Your revised manuscript is likely to be sent for further evaluation by all or a subset of the reviewers.

In addition to these revisions, you will need to complete some formatting changes, which you will receive in a follow up email. A member of our team will be in touch with a set of requests shortly.

We expect to receive your revised manuscript within 3 months. Please email us (plosbiology@plos.org) if you have any questions or concerns, or would like to request an extension.

At this stage, your manuscript remains formally under active consideration at our journal; please notify us by email if you do not intend to submit a revision so that we may withdraw it.

**IMPORTANT - SUBMITTING YOUR REVISION**

Your revisions should address the specific points made by each reviewer. Please submit the following files along with your revised manuscript:

1. A 'Response to Reviewers' file - this should detail your responses to the editorial requests, present a point-by-point response to all of the reviewers' comments, and indicate the changes made to the manuscript.

*NOTE: In your point-by-point response to the reviewers, please provide the full context of each review. Do not selectively quote paragraphs or sentences to reply to. The entire set of reviewer comments should be present in full and each specific point should be responded to individually, point by point.

You should also cite any additional relevant literature that has been published since the original submission and mention any additional citations in your response.

2. In addition to a clean copy of the manuscript, please also upload a 'track-changes' version of your manuscript that specifies the edits made. This should be uploaded as a "Revised Article with Changes Highlighted" file type.

*Re-submission Checklist*

When you are ready to resubmit your revised manuscript, please refer to this re-submission checklist: https://plos.io/Biology_Checklist

To submit a revised version of your manuscript, please go to https://www.editorialmanager.com/pbiology/ and log in as an Author. Click the link labelled 'Submissions Needing Revision' where you will find your submission record.

Please make sure to read the following important policies and guidelines while preparing your revision:

*Published Peer Review*

Please note while forming your response, if your article is accepted, you may have the opportunity to make the peer review history publicly available. The record will include editor decision letters (with reviews) and your responses to reviewer comments. If eligible, we will contact you to opt in or out. Please see here for more details:

https://blogs.plos.org/plos/2019/05/plos-journals-now-open-for-published-peer-review/

*PLOS Data Policy*

Please note that as a condition of publication PLOS' data policy (http://journals.plos.org/plosbiology/s/data-availability) requires that you make available all data used to draw the conclusions arrived at in your manuscript. If you have not already done so, you must include any data used in your manuscript either in appropriate repositories, within the body of the manuscript, or as supporting information (N.B. this includes any numerical values that were used to generate graphs, histograms etc.). For an example see here: http://www.plosbiology.org/article/info%3Adoi%2F10.1371%2Fjournal.pbio.1001908#s5

*Blot and Gel Data Policy*

We require the original, uncropped and minimally adjusted images supporting all blot and gel results reported in an article's figures or Supporting Information files. We will require these files before a manuscript can be accepted so please prepare them now, if you have not already uploaded them. Please carefully read our guidelines for how to prepare and upload this data: https://journals.plos.org/plosbiology/s/figures#loc-blot-and-gel-reporting-requirements

*Protocols deposition*

To enhance the reproducibility of your results, we recommend that if applicable you deposit your laboratory protocols in protocols.io, where a protocol can be assigned its own identifier (DOI) such that it can be cited independently in the future. Additionally, PLOS ONE offers an option for publishing peer-reviewed Lab Protocol articles, which describe protocols hosted on protocols.io. Read more information on sharing protocols at https://plos.org/protocols?utm_medium=editorial-email&utm_source=authorletters&utm_campaign=protocols

Thank you again for your submission to our journal. We hope that our editorial process has been constructive thus far, and we welcome your feedback at any time. Please don't hesitate to contact us if you have any questions or comments.

Sincerely,

Roli Roberts

Roland Roberts, PhD

Senior Editor

PLOS Biology

rroberts@plos.org

------------------------------------

REVIEWERS' COMMENTS:

Reviewer #1:

In this manuscript, the authors argue how plasmids (or mobile elements in general) could be studied through the lens of classic ecological theories, while also introducing an extended model to include multiple relevant processes acting on (the diversity of) plasmids. The manuscript is well written, and I enjoyed their perspective very much. There are a few points where I would argue the strength could be improved, or perhaps where I simply got confused.

First some broader, general remarks. I very much enjoyed the intro (very lovely written!) and the analysis of figure 1. However, in discussing figure 2 and 3 I feel like the authors are somewhat missing the opportunity to emphasise that these classical models (determinism vs. neutral) are a bit of a false dichotemy to begin with. Neutrality and selection are heavily variable under most reasonable conditions in nature, and indeed figure 4 reeled me back in by putting aside this black-and-white thinking. I found myself a little frustrated during the part of figure 2 and 3, which in the end wasn't needed as figure 4 brought all the nuance back. Perhaps some foreshadowing of what will be built towards could have avoided this frustration, which I think other readers may also experience.

One other general remark is that people have recently studied similar patterns in other MGEs (phages, IS elements, e.g. Flora Gaudilliere's work on transposons in prokaryotes https://www.biorxiv.org/content/10.1101/2025.07.22.666184v1.full.pdf+html), so I'd at least acknowledge that this way of thinking is already happening. That's not to say you're not doing something unique, but I would think it's better to frame this as a new way that people are thinking about this topic, rather than as a pioneering view.

Just a positive remark: that supplementary video is absolutely lovely, and it's illustrating a pretty cool result :)

Happy to look at your revised manuscript if the editor wishes, it's a really fun and light read :)

Some smaller points:

- Abstract: comma after "yet" missing. (double check me on this, I feel like it should be followed by a comma but I could be wrong)

- Personal preference, but in figure 1 caption / it's discussion in the text, I would personally not use "striking" here, as these patterns are indeed the default behaviour from neutral models (as you explain later), so any DEVIATION from this pattern would be striking. I would instead simply emphasise the similarity without any adjectives.

- At the bottom of page 4 "i.e. spread in the bacterial population, when alone exploiting..." this sentence structure is confusing. I would move the "alone" part forward in the sentence, e.g. "... each plasmid can grow when alone, i.e. spread through the bacterial population, in the absence of competition by the other." (or sth like that)

- Figure 3 A: why did you go to red-green when figure 2 had blue-red plasmids? I am red-green colour blind, so I appreciated the colours in 2 more.

- Figure 3 B: I would not use viridis-colours (a continuous scale) for displaying what I think are categorical differences of plasmid species.

- Figure 3 D: is the effect of dispersal monotonic? Is higher dispersal always leading to higher diversity, or can it also go down again? (I think not, as your model assumes only bacteria disperse while environments stay heterogeneous? perhaps a supplementary figure on how dispersal shapes diversity would be nice)

- I may have missed it but are the parameters choices mentioned anywhere else but in the supplementary file? Is the fit of the data shown in figure 3/4 using inferred parameters? Wasn't completely clear to me.

- Methods: I am not entirely sure why the vertical transmission term (Ei) looks so complicated with the log(alpha) stuff. It's probably obvious in hind sight but I can't see it now, perhaps make sure you explain why that choice was made / why it's biologically sound.

Reviewer #2:

In this manuscript, Tuffet and colleagues consider plasmid distributions using established ecological frameworks developed to understand animal and plants. They consider how plasmid 'species' distributions in a database of E. coli genomes mirrors those of animals and plants, and develop a general mathematical model of ecological processes. The model yields results consistent with these patterns, with the main conclusion highlights the role that neutral processes could play in determining plasmid distributions. Overall this was a nicely written and presented paper. The key result — that neutral models do a reasonable job of capturing patterns of plasmid distribution — is valuable and somewhat provocative, since many people assume that plasmids are driven principally by selection for specific accessory traits. More than this, the application of these ecological frameworks to mobile genetic elements dynamics is pioneering and makes a valuable contribution to the literature, and an exemplar for future studies in this area. Even if the model is relatively simple, and the key results somewhat obvious, I think this work has the potential to make an important impact. However, I have some major concerns with the way that the data are interpreted, principally owing to the biases in the plasmid database.

Main issues/comments:

- The work focuses on E. coli and its cognate plasmids, and generates its database using E. coli genomes from NCBI RefSeq. Focusing on a single species is sensible, but I'm concerned that the sampling of strains that ultimately yield the genomes on RefSeq will have been highly biased towards human and animal pathogens, resulting in repeating sampling of epidemic clones. For example, ST131 is a dominant pandemic clone that frequently harbours plasmids. Consequently, I can envisage how this kind of biased sampling might produce a species abundance distribution of the kind described in Figure 1A simply as a consequence of a particular pathogenic clone being more predisposed to a particular plasmid or PTU, and 'rarer' plasmids being found fewer times just because their hosts are less of a concern (and therefore less likely to be sequenced) than pathogens. There have been some attempts to control for this kind of correlation, which the authors might consider, e.g. as described by Dewar et al. 2021 10.1038/s41559-021-01573-2. Another approach (perhaps less robust) would be to downsample their data to a single clone per BioProject, to ensure that their results are not dominated by repeated sampling of a clonal outbreak. But the authors need to make some effort to demonstrate that biased sampling in the E. coli dataset is not the driver of their observations.

- How are the models parameterised? The authors include tables of values (Tables S1-3) but these are not well described or justified. I'm not sure whether the regimes presented are ecologically relevant, nor am I sure of the sensitivity of the results to the selected parameters. There appears to be some sensitivity analysis in Figs. S2 and S3 but these are not well described. More detail needs to be provided here, and an additional column in the table of parameter values justifying the selected range for each term with reference to the literature.

- The first conclusion in the results is that plasmid abundance patterns 'mirror' those of animals and plants, a conclusion that is based on visual inspection of rank (log) abundance curves. It is a bit unsatisfying to rest the conclusion on this somewhat subjective 'eyeball' analysis. It would be valuable to formally test how similar these curves are. This might be done with a two-one-sided test variant of the Kolmogorov-Smirnov test, some kind of bootstrapping/permutation test, or there may be a more elegant approach that I am not familiar with.

Minor comments:

- Figure S1 panels C and D are difficult to interpret. More information should be provided in the legend to explain what these are showing.

- On the subject of selection vs. neutral processes in plasmid abundance patterns, the authors might like to touch on the recent paper by Adrian Cazares and colleagues (2025) that describes plasmids from pre-antibiotic era strains, which suggests a greater diversity of plasmids from the pre-antibiotic era. I wonder how SADs would be affected by strong selection on a selected subset of plasmids?

- It would be interesting also to consider how plasmid abundance patterns are affected by the possibility of resistance gene capture, i.e. where a plasmid is lost and the resistance genes migrate to the chromosome. Perhaps this is similar to a very low conjugation rate? Might be worth a couple of words in the discussion, since 'chromosomal capture' is a key principle of the plasmid paradox.

Reviewer #3:

In the manuscript "Plasmid diversity and dynamics in the light of ecological theories", the authors argue that plasmids can be viewed as ecological entities competing for bacterial hosts. This is a compelling perspective that could have important implications for how we understand plasmid persistence, diversity, and their role in bacterial evolution, including the spread of antibiotic resistance. However, earlier theoretical studies have explored this perspective in various forms for decades, and contemporary work continues to refine it. For example:

10.1086/416582

10.1007/BF02540223

10.1038/nrmicro1643

10.1016/j.ecolmodel.2025.111418

10.48550/arXiv.2509.01695

The manuscript is explicitly framed as a bridge between plasmid biology and ecological theory. This interdisciplinary ambition is appealing, but it also raises the question of what genuinely new insight is provided to either community. From a plasmid biology perspective, the ecological framing is elegant but largely rearticulates mechanisms that have been extensively explored in prior theoretical and empirical work, including competition, trade-offs between horizontal and vertical transmission, environmental heterogeneity, stochastic drift, dispersal, and innovation. As such, the study does not clearly yield new plasmid-specific mechanisms or actionable predictions that would change how plasmid biologists think about plasmid persistence, diversity, or resistance evolution.

From an ecological perspective, the work primarily applies well-established concepts, including neutral and niche theory, emergent neutrality, species abundance distributions, and metacommunity dynamics, to plasmid systems. While plasmids provide an interesting and tractable biological example, the manuscript does not appear to extend, challenge, or refine ecological theory itself, but rather illustrates its applicability in a new domain. Consequently, it is unclear whether ecologists would view this study as offering conceptual advances, as opposed to a compelling case study.

In this sense, the manuscript reads primarily as a conceptual synthesis that successfully reframes existing ideas across disciplines, but does not yet clearly deliver new biological insight for plasmid research nor new theoretical insight for ecology. Clarifying what is genuinely learned on either side of this interface, beyond the demonstration that ecological frameworks can be applied to plasmids, would substantially strengthen the contribution. Absent such clarification, the work may be better suited for a more ecology-focused or conceptual journal, where its integrative perspective would be appreciated as synthesis rather than as a primary biological advance.

# Author comment: Author response to Decision Letter 2

**DOI:** 10.1371/journal.pbio.3003918.r003

5 May 2026

Attachment Submitted filename: Response to Reviewers.pdf

# Peer review: Decision Letter 2

**DOI:** 10.1371/journal.pbio.3003918.r004

**Authors:** Roland Roberts

8 Jun 2026

Dear Dr Tuffet,

Thank you for your patience while we considered your revised manuscript "Plasmid diversity and dynamics in the light of ecological theories" for publication as a Short Report at PLOS Biology. This revised version of your manuscript has been evaluated by the PLOS Biology editors, the Academic Editor and the original reviewers.

Based on the reviews, we are likely to accept this manuscript for publication, provided you satisfactorily address the remaining points raised by the reviewersand the following data and other policy-related requests.

IMPORTANT - please attend to the following:

a) We need the Title to include an active verb. We suggest changing it to: "Ecological theory illuminates plasmid diversity and dynamics"

b) Please attend to the remaining requests from the reviewers. You'll see that reviewer #1 is now happy, but wants you to change the colour scale in one Figure. Reviewer #2 is mostly satisfied, but thinks that two of your parameters are somewhat extreme (high transmission, easy loss), so would like you to check the sensitivity to other values of these parameters. Reviewer #3 is now happier than s/he was, but remains somewhat sceptical about the magnitude of the advance.

c) Many thanks for providing the data and code. Please cite the location of the data clearly in all relevant main and supplementary Figure legends (i.e. Figs 1-4, Figs S1-S4), e.g. “The data and code required to generate this Figure can be found in https://zenodo.org/records/19951374"

As you address these items, please take this last chance to review your reference list to ensure that it is complete and correct. If you have cited papers that have been retracted, please include the rationale for doing so in the manuscript text, or remove these references and replace them with relevant current references. Any changes to the reference list should be mentioned in the cover letter that accompanies your revised manuscript.

In addition to these revisions, you may need to complete some formatting changes, which you will receive in a follow up email. A member of our team will be in touch with a set of requests shortly. If you do not receive a separate email within a few days, please assume that checks have been completed, and no additional changes are required.

We expect to receive your revised manuscript within two weeks.

To submit your revision, please go to https://www.editorialmanager.com/pbiology/ and log in as an Author. Click the link labelled 'Submissions Needing Revision' to find your submission record. Your revised submission must include the following:

- a cover letter that should detail your responses to any editorial requests, if applicable, and whether changes have been made to the reference list

- a Response to Reviewers file that provides a detailed response to the reviewers' comments (if applicable, if not applicable please do not delete your existing 'Response to Reviewers' file.)

- a track-changes file indicating any changes that you have made to the manuscript.

NOTE: If Supporting Information files are included with your article, note that these are not copyedited and will be published as they are submitted. Please ensure that these files are legible and of high quality (at least 300 dpi) in an easily accessible file format. For this reason, please be aware that any references listed in an SI file will not be indexed. For more information, see our Supporting Information guidelines:

https://journals.plos.org/plosbiology/s/supporting-information

*Published Peer Review History*

Please note that you may have the opportunity to make the peer review history publicly available. The record will include editor decision letters (with reviews) and your responses to reviewer comments. If eligible, we will contact you to opt in or out. Please see here for more details:

https://plos.org/published-peer-review-history/

*Press*

Should you, your institution's press office or the journal office choose to press release your paper, please ensure you have opted out of Early Article Posting on the submission form. We ask that you notify us as soon as possible if you or your institution is planning to press release the article.

*Protocols deposition*

To enhance the reproducibility of your results, we recommend that if applicable you deposit your laboratory protocols in protocols.io, where a protocol can be assigned its own identifier (DOI) such that it can be cited independently in the future. Additionally, PLOS ONE offers an option for publishing peer-reviewed Lab Protocol articles, which describe protocols hosted on protocols.io. Read more information on sharing protocols at https://plos.org/protocols?utm_medium=editorial-email&utm_source=authorletters&utm_campaign=protocols

Please do not hesitate to contact me should you have any questions.

Sincerely,

Roli Roberts

Roland Roberts, PhD

Senior Editor

rroberts@plos.org

PLOS Biology

------------------------------------------------------------------------

REVIEWERS' COMMENTS:

Reviewer #1:

[identifies himself as Bram van Dijk]

Dear authors,

I am happy with the revisions, and happy to see this work published. I did however submit 'minor revisions' as I still would like one of my suggestions to be followed through (but it remains a suggestion). I would still consider is changing the colour scale of 3B from viridis to randomised colours. There are plenty of packages available to do this. There is no order in the species data, so assigning colours along a continuous scale is really a bad choice in my opinion.

Reviewer #2:

The authors have responded satisfactorily to all my suggestions and comments, and I'm very glad to see the deeper analyses controlling for potential outbreak clones — this is very nice.

However, now that they have provided full details about model parameterisation I'm afraid to say have a new query that I'd like reassurance on:

- The authors state that their vertical transmission rate was set to 0.9, meaning there's a 10% chance of losing the plasmid each generation. This seems high when considering the literature and e.g. values in the provided citation, particularly when considering most natural transmissible plasmids have partitioning apparatus and toxin-antitoxin systems. The Lau et al. 2013 paper suggests that rates of loss are often as low as 1e-6. Are the main findings robust to higher vertical transmission rates?

- Similarly, the use of horizontal transmission rates between 1e-5 and 1e-7 is at the higher end of those indicated in the provided references, which indicate plasmids frequently have transmission rates 1e-14 to 1e-10. How robust are the results to (much) lower horizontal transfer rates?

I don't think the values chosen by the authors are unreasonable, and I appreciate it can be hard to match up real-world measurements with abstract model formulations. It would be good to know, though, that the authors observations do not just emerge from a peculiar regime of highly transmissible, easily-lost plasmids.

Minor comments/issues:

- Methods section. Section on plasmid diversity could be clarified (line 502): "We filtered all complete E. coli, Klebsiella, and Salmonella genomes available on NCBI RefSeq…". Later in the paragraph (line 507): "We extracted a total of 6,131 (E. coli), 5,149 (Klebsiella) and 1,517 (Salmonella) plasmids for downstream analysis." Similar later in the paragraph (line 510)

- Overlapping labels in Figure 1.

- Figure 3C guide does not match what's described in the legend.

- RAC abbreviation introduced more than once (line 133, 355, 410) — this isn't necessarily a problem though, it's often nice to be reminded what acronyms stand for!

- Line 960 typo (Escherichai); line 965 (Kelbsiella)

- Line 971. Is text missing from this sentence (e.g. between the brackets, at the end of the sentences)? It appears correctly in the .docx but not in the .pdf. Also on line 975.

- Total bacterial density parameter not present in the table S2. Is this an error?

Reviewer #3:

The authors have substantially improved the manuscript and have clarified the scope of the contribution. The revised version no longer presents the ecological analogy itself as the main novelty. Instead, it more clearly focuses on plasmid assemblage diversity and on the quantitative fit between ecological models and plasmid rank-abundance patterns. The strongest biological contribution is the proposed community-assembly framework for interpreting plasmid pangenomes. I also appreciate the authors' effort to identify a more concrete biological implication of the model: that strong antibiotic pressure may accelerate competitive exclusion and reduce the diversity of rare plasmids. However, I do not view this as a fundamentally new prediction, since it follows directly from standard ecological expectations under strong directional selection. Its value lies more in connecting this expectation to plasmid pangenome data than in proposing a new mechanism.

Overall, the manuscript is clearer and stronger than the previous version. Nevertheless, I still view its novelty as moderate, since the main contribution lies in integrating and applying existing ecological concepts to plasmid assemblages rather than in developing a fundamentally new biological principle.

# Author comment: Author response to Decision Letter 3

**DOI:** 10.1371/journal.pbio.3003918.r005

6 Jul 2026

Attachment Submitted filename: Response_to_Reviewers_auresp_3.pdf

# Editor report: Decision Letter 3

**DOI:** 10.1371/journal.pbio.3003918.r006

**Authors:** Roland Roberts

8 Jul 2026

Dear Dr Tuffet,

Thank you for the submission of your revised Short Report "Ecological theory sheds light on plasmid diversity and dynamics" for publication in PLOS Biology. On behalf of my colleagues and the Academic Editor, Sara Mitri, I'm pleased to say that we can in principle accept your manuscript for publication, provided you address any remaining formatting and reporting issues. These will be detailed in an email you should receive within 2-3 business days from our colleagues in the journal operations team; no action is required from you until then. Please note that we will not be able to formally accept your manuscript and schedule it for publication until you have completed any requested changes.

Please take a minute to log into Editorial Manager at http://www.editorialmanager.com/pbiology/, click the "Update My Information" link at the top of the page, and update your user information to ensure an efficient production process.

PRESS: We frequently collaborate with press offices. If your institution or institutions have a press office, please notify them about your upcoming paper at this point, to enable them to help maximise its impact. If the press office is planning to promote your findings, we would be grateful if they could coordinate with biologypress@plos.org. If you have previously opted in to the early version process, we ask that you notify us immediately of any press plans so that we may opt out on your behalf.

We also ask that you take this opportunity to read our Embargo Policy regarding the discussion, promotion and media coverage of work that is yet to be published by PLOS. As your manuscript is not yet published, it is bound by the conditions of our Embargo Policy. Please be aware that this policy is in place both to ensure that any press coverage of your article is fully substantiated and to provide a direct link between such coverage and the published work. For full details of our Embargo Policy, please visit http://www.plos.org/about/media-inquiries/embargo-policy/.

Thank you again for choosing PLOS Biology for publication and supporting Open Access publishing. We look forward to publishing your study.

Sincerely,

Roli Roberts

Roland G Roberts, PhD, PhD

Senior Editor

PLOS Biology

rroberts@plos.org
